# Molecular-genetic profiling and high-throughput *in vitro* drug screening in NUT midline carcinoma—an aggressive and fatal disease

**DOI:** 10.18632/oncotarget.22862

**Published:** 2017-12-02

**Authors:** Anja Stirnweiss, Joyce Oommen, Rishi S. Kotecha, Ursula R. Kees, Alex H. Beesley

**Affiliations:** ^1^ Leukaemia and Cancer Genetics Program, Telethon Kids Institute, The University of Western Australia, Perth, Australia; ^2^ Drug Discovery Group, Telethon Kids Institute, The University of Western Australia, Perth, Australia; ^3^ Department of Haematology and Oncology, Princess Margaret Hospital for Children, Perth, Australia; ^4^ School of Paediatrics and Child Health, University of Western Australia, Perth, Australia

**Keywords:** NUT midline carcinoma, bromodomain inhibitor, exome sequencing, drug screen, DNA-repair

## Abstract

NUT midline carcinoma (NMC) is a rare and aggressive cancer, with survival typically less than seven months, that can arise in people of any age. Genetically, NMC is defined by the chromosomal fusion of *NUTM1* with a chromatin-binding partner, typically the bromodomain-containing protein *BRD4*. However, little is known about other genetic aberrations in this disease. In this study, we used a unique panel of cell lines to describe the molecular-genetic features of NMC. Next-generation sequencing identified a recurring high-impact mutation in the DNA-helicase gene *RECQL5* in 75% of lines studied, and biological signals from mutation-signature and network analyses consistent with a general failure in DNA-repair. A high-throughput drug screen confirmed that microtubule inhibitors, topoisomerase inhibitors and anthracyclines are highly cytotoxic in the majority of NMC lines, and that cell lines expressing the *BRD4-NUTM1* (exon11:exon2) variant are an order of magnitude more responsive to bromodomain inhibitors (iBETs) on average than those with other *BRD4-NUTM1* translocation variants. We also identified a highly significant correlation between iBET and aurora kinase inhibitor efficacy in this study. Integration of exome sequencing, transcriptome, and drug sensitivity profiles suggested that aberrant activity of the nuclear receptor co-activator *NCOA3* may correlate with poor response to iBETs. In conclusion, our data emphasize the heterogeneity of NMC and highlights genetic aberrations that could be explored to improve therapeutic strategies. The novel finding of a recurring *RECQL5* mutation, together with recent reports of chromoplexy in this disease, suggests that DNA-repair pathways are likely to play a central role in NMC tumorigenesis.

## INTRODUCTION

NUT midline carcinoma (NMC), also known as NUT carcinoma, is an invariably fatal malignancy with an average survival time of less than 7 months [1]. The tumors typically arise in the mediastinum and upper aerodigestive track, and present as extremely aggressive undifferentiated carcinomas, with or without squamous differentiation [2]. Data collected retrospectively through the International NMC Registry (http://www.nmcregistry.org) have shown that conventional chemotherapeutic drugs have no positive effect on disease progression and survival [3]. The disease is driven by *NUTM1*-fusion oncogenes that disrupt cellular differentiation. While little is known about the cellular role of *NUTM1*, the *NUTM1*-partner genes (e.g. *BRD4*, *BRD3, NSD3*) are recognized as master regulators of chromatin structure and function. Recent studies have shown that the NUTM1 component of NMC fusion proteins can recruit histone acetyltransferases, such as p300 and CREB-binding protein [4–6], whilst the bromodomain moieties of BRD4 (or BRD3) bind to acetylated histones. In this way, NUTM1-fusion proteins induce histone hyperacetylation at defined chromatin sites, thus inactivating genes required for apoptosis and differentiation through the sequestration of p300 [5–8]. However, ChIP-Seq data have revealed little overlap in acetylated chromatin domains bound by BRD4-NUTM1 in different NMC samples, with the only consistently affected loci being those of *MYC* and *TP63* [7]. In keeping with this observation, knock-down experiments have demonstrated that these two genes are important for maintaining the aggressive phenotype of NMC [7, 9].

Recognition of the importance of *BRD4* in cancer has led to the development of a new generation of anti-cancer compounds that specifically target the BET (bromodomain and extra-terminal motif) family of proteins, of which *BRD3* and *BRD4* are key members [10–13]. Importantly, it is thought that these bromodomain inhibitors (iBETs) may also directly target the BRD4/3-NUTM1 fusion proteins expressed in NMC. By studying samples expressing the variant fusion NSD3–NUTM1, French *et al*. have shown that a key component of the oncogenic mechanism in the majority, if not all NMC tumors, is the formation of an iBET-sensitive complex involving NSD3, BRD4 and NUTM1 [14]. As a result of significant pre-clinical responses to these drugs, Phase I/II clinical trials have been opened to investigate the efficacy of different iBETs in NMC and other advanced cancers (Clinical Trial Identifiers: NCT01587703, NCT02307240, NCT01987362, NCT02711137, NCT02431260, and NCT02259114; Supplementary Table 1). Results from these trials are pending, but indications from a report describing survival times of more than double the current median in three out of four NMC patients receiving treatment with the iBET OTX-015, are promising [15].

Despite this preliminary report and a wealth of encouraging laboratory data, several pre-clinical studies and clinical trials have indicated that the therapeutic benefit of iBETs may be limited by toxicity at higher doses, and by the acquisition of resistance [15–20]. Two of these studies reported an activation of the WNT pathway in iBET-resistant acute myeloid leukemia that was associated with the promotion of a stem cell-like phenotype [17, 18]. Triple-negative breast cancer cells on the other hand, have been shown to acquire resistance through the binding of BRD4 to the transcriptional activator MED1 and the subsequent activation of *MYC*; [19] whilst in colorectal cancer cells, loss of TRIM33 and subsequent activation of the TGF-β receptor signaling have been implicated [20]. Such studies highlight the potential diversity of iBET resistance mechanisms in tumors.

We previously demonstrated that the efficacy of BET-inhibition in NMC might vary in regard to either the precise cell of origin of each tumor, or the specific chromosomal translocation involved [21]. In the present study, we have performed whole exome and transcriptome sequencing on a large panel of NMC cell lines to comprehensively describe the molecular-genetic landscape of NMC, a critical step towards developing novel therapy approaches for this aggressive disease.

## RESULTS

### Overview of NMC cell line features

The rarity of NMC significantly limits the availability of tumor material, thus tumor-derived cell lines provide an invaluable resource for further research. In this study, we compared the drug response profiles and the genetic features of 12 NMC cell lines (HCC-2429 [22], PER-403 [23], PER-624 [24], PER-704 [21], P896-CL [25], TC797 [26], TY82 [27], RPMI2650 [28], 8645 [29], 10326 [30], 11060 [10] and 14169 [31]). This cell line panel is representative for all known *BRD4-NUTM1* and *BRD3-NUTM1* fusion variants, with the majority of the lines having either a *BRD4-NUTM1 ex11:ex2,* or a *BRD4-NUTM1 ex15:ex2* breakpoint (Supplementary DataFile 1). The characteristics of the patients from whom the cell lines were derived are reflective of NMC patient demographics [3] in regard to both gender distribution and age range (8 to 52 years; Supplementary DataFile 1). We identified one of the cell lines, RPMI2650, using expression data from the Genomics in Drug Sensitivity in Cancer Project [32, 33] (by screening for positive expression of *NUTM1*) and verified that this line indeed carries an NMC breakpoint. This finding was independently confirmed in another publication whilst conducting this study [34]. The discovery of this NMC cell line languishing in a public repository has two implications: (i) it emphasizes the concept that expression of *NUTM1* in an undifferentiated carcinoma is diagnostic for NMC, and (ii) it reinforces the fact that NMC has historically been under-diagnosed, highlighting the potential for further discovery of samples in bio-banks around the world.

### Drug-sensitivity profile of the carcinoma cell line panel

**Figure 1:**
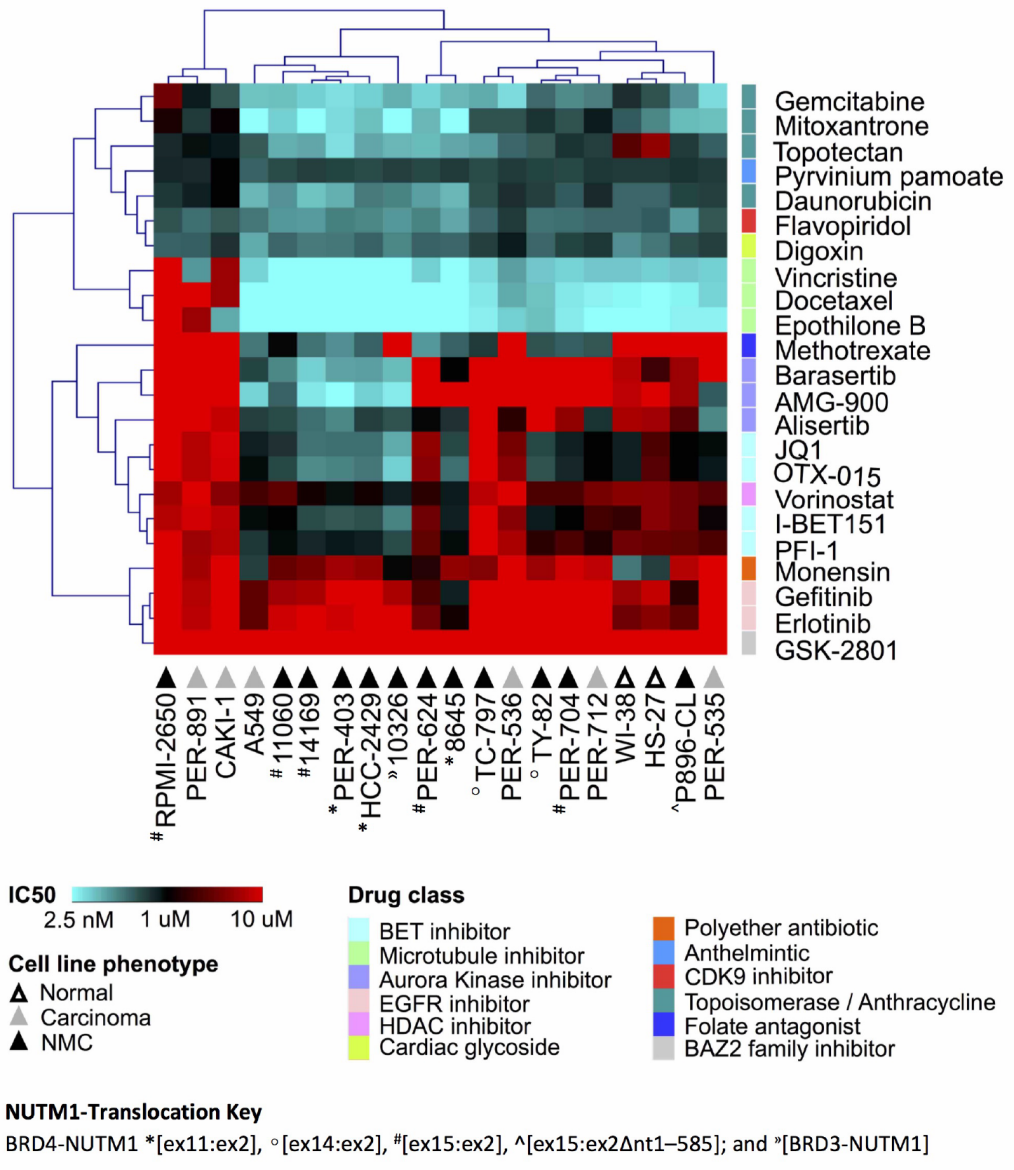
Drug response profile of the carcinoma cell line panel Unsupervised hierarchical clustering of selected anti-cancer agents, based on the concentration that is cytotoxic for 50% of the cells (IC50) in two non-disease fibroblasts (‘normal’, white triangles), six non-NMC carcinoma (‘carcinoma’, grey triangles) and 12 NMC lines (‘NMC’, black triangles). The different NUTM1-fusion variants expressed in those 12 NMC cell lines are indicated with the following prefixes: BRD4-NUTM1 ^*^[ex11:ex2], °[ex14:ex2], ^#^[ex15:ex2], ^^^[ex15:ex2Δnt1–585]; and ^»^[BRD3-NUTM1]. Multiple drug classes are represented as indicated by the color key.

We previously reported that NMC cells have considerable variability in the response to certain drug classes, including iBETs [21]. Since those original observations were limited to a small number of cell lines, we selected a shortlist of compounds with good efficacy in our previous study, together with a number of additional compounds with known relevance for NMC, for further analysis in a more comprehensive cell line panel. This panel included the 12 NMC cell lines described above, plus six carcinoma lines of non-NMC origin and two non-disease (i.e. nominally non-cancer or ‘normal’) fibroblast lines (Supplementary DataFile 1). Two of the tested drugs were iBETs (JQ1, I-BET151) for which we have reported marked differences in efficacy in NMC cell lines of distinct genetic background [21]. We included two additional iBETs in the present study (PFI-1, OTX-015) to confirm this observation and determine if such differences might relate to this drug class in general, rather than to specific compounds. Due to the known relationship between BRD4 and aurora kinases (AURK), we included three AURK inhibitors (iAURKs) in the screen (barasertib, AMG-900 and alisertib) [35, 36]. In line with our previous findings [21], we were able to confirm that anthracyclines (e.g. daunorubicin), topoisomerase inhibitors (e.g. topotecan, gemcitabine, mitoxantrone) and microtubule poisons (e.g. docetaxel, vincristine, epothilone B) were the most consistently cytotoxic drug classes across the cell line panel, while the efficacy of iAURKs and iBETs varied considerably (Figure 1 and Supplementary DataFile1). However, there was no clear pattern of drug-response that could be delineated by cell phenotype (i.e. between NMC, non-NMC carcinoma and ‘normal’ fibroblast lines), demonstrating the overriding importance of cellular context for determining cytotoxic responses and the difficulty of selecting appropriate agents for precision medicine. The WNT-pathway inhibitor pyrvinium pamoate was consistently effective at nanomolar doses (ranging from 95-906 nM), whilst the folate antagonist methotrexate, a drug used most typically in the treatment of hematological malignancies, showed surprisingly good efficacy in a subset of cell lines, although again this was not specific to NMC (Figure 1).

**Figure 2:**
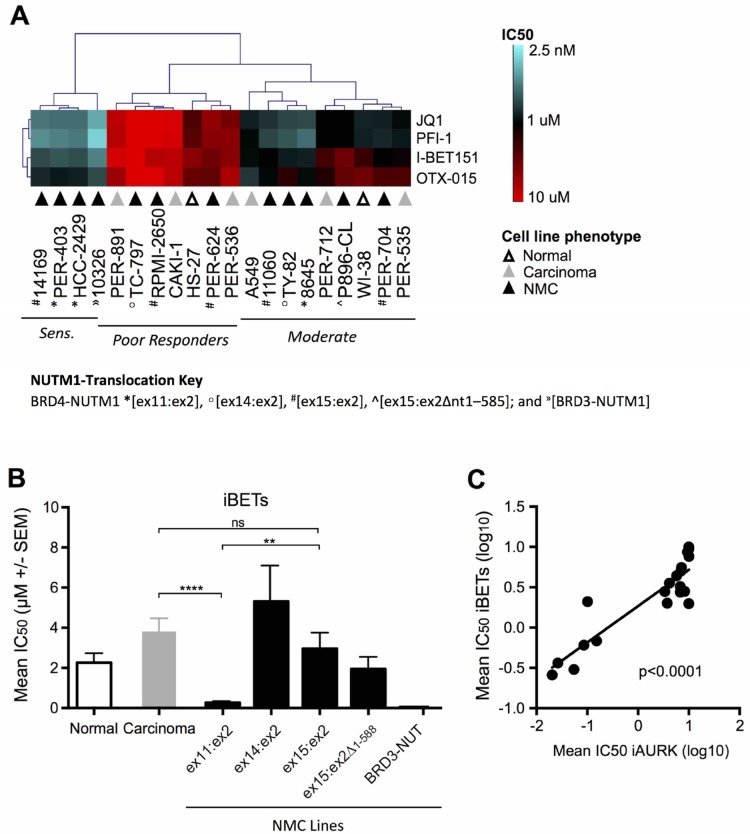
Comparative efficacy of iBET compounds in the carcinoma cell line panel (**A**) Unsupervised hierarchical clustering of iBET IC50 values across non-disease fibroblasts (‘normal’, white triangles), non-NMC carcinoma (‘carcinoma’, grey triangles) and NMC cell lines (‘NMC’, black triangles; BRD4-NUTM1 ^*^[ex11:ex2], °[ex14:ex2], ^#^[ex15:ex2], ^^^[ex15:ex2Δnt1–585]; and ^»^[BRD3-NUTM1]), with lines classified as sensitive, moderate or poor-responders. (**B**) Combined mean of JQ1, PFI-1, I-BET151, and OTX-015 IC50 values (μM ± SEM) in normal (white bars), non-NMC carcinoma (grey bars), and NMC cell lines (black bars). NMC cell lines were further divided based on the *NUTM1*-fusion variant expressed; for *BRD4-NUTM1* translocated cell lines, labeling refers to the transcript breakpoint position identified in each case (e.g. ex11:ex2 indicates that *BRD4* exon 11 is fused to *NUTM1* exon 2); ^**^p < 0.01; ^****^p < 0.0001; ns, not significant (unpaired *t*-test for groups with *n* ≥ 3). (**C**) Linear regression analysis of mean iBET IC50 (JQ1, PFI-1, I-BET 151, and OTX-015 combined) and mean aurora kinase inhibitor IC50 (iAURK: alisertib, barasertib, and AMG-900 combined) for all cell lines in the panel (i.e. normal, non-NMC carcinoma, and NMC).

Subsequent unsupervised hierarchical clustering focusing solely on iBET responses across the cell line panel, identified three distinct groups of iBET sensitivity (Figure 2A), which we refer to herein as ‘sensitive’, ‘moderate responders’ and ‘poor responders’. While the moderate (*n* = 9) and poor responder (*n* = 7) groups were comprised of cell lines from all three phenotypes (i.e. non-disease fibroblasts, non-NMC carcinoma and NMC), only NMC lines (*n* = 4) were represented in the sensitive group (14169, PER-403, HCC2429 and 10326). When further segregated based on NMC gene-fusion subtype (Figure 2B), iBET treatment was significantly more effective in NMC cell lines expressing the *BRD4-NUTM1 ex11:ex2* variant compared to those with a *BRD4-NUTM1 ex15:ex2* fusion (*p* < 0.01) or non-NMC carcinomas (*p* < 0.0001). It is notable that the one cell line carrying a *BRD3-NUTM1* translocation (10326) was also highly sensitive to iBET treatment. A similar response pattern was observed for the iAURKs, with analysis confirming a highly significant correlation between iAURK and iBET sensitivities (Figure 2C, *p* < 0.0001). This observation is consistent with the findings of a recent report describing a relationship between iBET treatment and the suppression of AURKs [37]. Although the number of lines in each of the sub-groups shown in Figure 2B is relatively small, the data suggest that the exact type of breakpoint expressed in NMC tumors may affect the efficacy of iBETs and iAURKs. The finding of differential sensitivity based on underlying biology has significant clinical implications, something that will be important to prospectively assess during ongoing clinical trials.

### Potential germline variants in the primary patient sample P896

Due to the scarcity of NMC, and its historical under-diagnosis, matched tumor-normal samples for next-generation sequencing analysis are rare. However, from a previously described NMC patient (P896) [25] we were able to derive an early passage fibroblast line (P896-FB) that could be used as a normal (non-tumor, or constitutive) control for this sample. Absence of any *NUTM1* translocation in P896-FB was confirmed by both genomic PCR, and RT-PCR, and the karyotype was normal (Supplementary DataFile 1). We thus performed Illumina–based whole exome sequencing (WES) of P896 and P896-FB and analyzed the data using GATK [38] to identify germline variants that could potentially have contributed to the development of the disease in this patient (see Supplementary Materials). A total of 179 rare variants with the potential for functional impact were identified (169 SNPs, 10 indels), that were common to P896 and P896-FB but absent in normal human blood samples downloaded from a previously published study [39]. Of those 179 variants, only 13 were annotated by Variant Effect Predictor (VEP, from Ensembl) [40] as having high functional impact (Supplementary DataFile 2). Although it is impossible to draw definitive conclusions in regard to the potential biological significance of germline features from a single patient sample, there were a number of features of particular interest amongst the 179 variants (Supplementary DataFile 2). Several variants were observed in genes involved in histone and chromatin modification, including *HIST1H1A*, *KDM1B*, *BAHD1* and *SRCAP*, the latter known to have direct interactions with BRD4. There were also variants affecting the NOTCH-signaling pathway (*FLT3*, *FZD9* and *NOTCH1*), which plays an important role in cellular differentiation. Finally, there were variants in genes involved in DNA-damage response and apoptosis (*BECN1*, *CCAR1*, *TP53BP1*), as well as control of chromosome-separation during cell division (*INSC*, *SYCE3*). The latter observation is of particular interest in the context of a disease for which the driving oncogenic feature is a chromosomal translocation event.

As a final comparison, we ran the same GATK pipeline to call variants from WES data obtained from 11 of the NMC cell lines, however very few of these P896 germline candidates (only 10 out of 179) were found to be present in any of these lines, and there were none that were present in more than one NMC cell line (Supplementary DataFile 2), arguing against the involvement of highly recurrent germline events in the tumorigenesis of NMC.

### Genetic landscape of NMC

**Figure 3:**
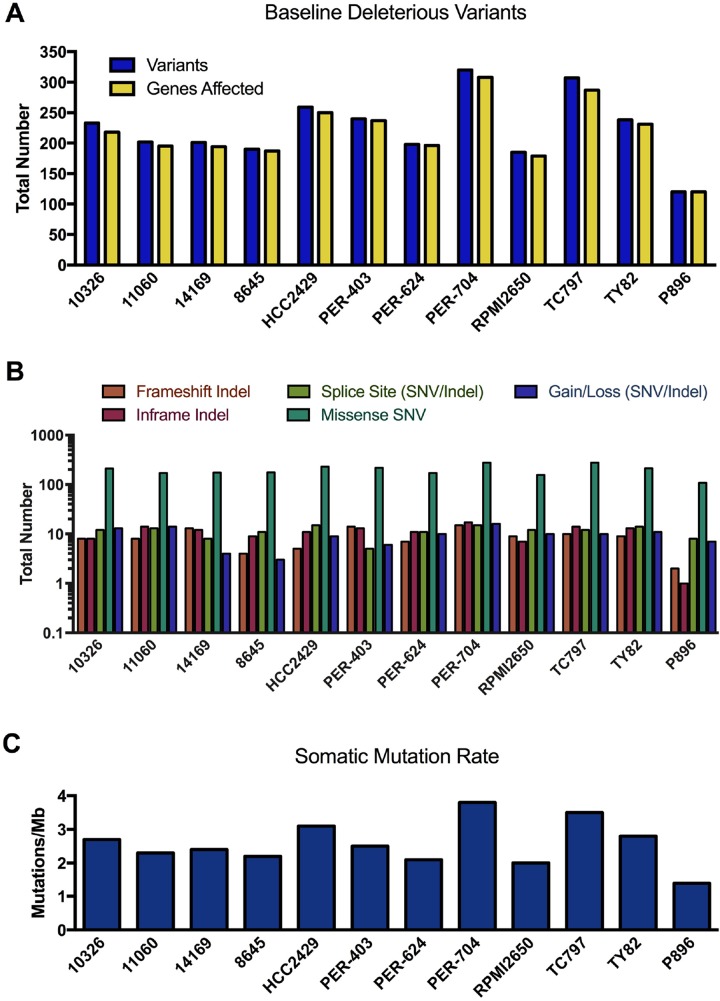
Mutational landscape of NMC samples (**A**) Total number of rare variants with potential functional consequences (baseline deleterious variants), and the number of genes affected by those variants per sample. (**B**) Coding sequence consequences of these baseline deleterious variants. (**C**) The somatic mutation rate across exome target regions in NMC samples.

The fibroblast line P896-FB was then used as a control to call somatic mutations in the primary NMC sample P896 using two different paired tumor-normal algorithms, MuTect analysis [41] (which calls SNVs only) and Strelka [42] (which calls both SNVs and indels). To call somatic mutations in the NMC cell lines (for which matched constitutive samples were simply not available), we again ran MuTect and Strelka using P896-FB as the non-tumor comparator, since the germline comparison with P896 (Supplementary DataFile 2) had already identified any variants that would otherwise be overlooked in this approach. The use of P896-FB as a comparator has the additional advantage of filtering out non-specific variation associated with cell culturing, as well any systematic artefacts specific to the exome sequencing platform used in this study. However, in the absence of constitutive samples for each of the NMC lines, it is probable that a considerable proportion of these ‘raw’ MuTect/Strelka variants will be germline rather than somatic. We thus annotated these using VEP and performed a conservative filtering process to identify rare variants (i.e. those with minor allele frequencies < 1% across all ExAC, 1000 Genome and dbSNP146 populations), and further restricted the list to those in protein-coding genes and predicted to have at least a moderate probability of biological impact (full details provided in Supplementary Materials). We refer to these as ‘baseline deleterious variants’, of which there were an average of 234 identified in the NMC cell lines, with fewer (120 variants) being observed in the primary NMC specimen P896 (Figure 3A). The number of unique genes carrying these mutations in each sample was similar to the actual number of unique variants, with each gene thus typically affected by only a single mutation (full list provided in Supplementary DataFile 3). The functional effect of these baseline deleterious variants (gain/loss, missense, frameshift etc.) is shown in Figure 3B.

The somatic mutation rate calculated for NMC cell lines ranged from 2.0–3.8 mutations/Mb (Figure 3C), whilst the rate for P896 was 1.4 mutations/Mb. These rates sit at the lower end of the spectrum for somatic mutations observed in other cancer types, which range from 0.001–400 mutations/Mb [43]. It is known that certain childhood cancers such as acute lymphoblastic leukemia carry the lowest rates of somatic mutation, whilst those in older patients or which are related to chronic mutagenic exposure (e.g. tobacco smoking), have the highest [44]. NMC is a disease that affects people of any age, with the cell lines used in the present study derived from patients aged between 8 and 52 years (Supplementary DataFile 1), and can arise in a variety of organs and tissues (including lung and larynx). Hence, the somatic mutation estimates for these samples will reflect the diversity in the presentation of the disease and the varied potential for exposure to mutagenic processes.

### Mutational signatures associated with NMC

**Figure 4:**
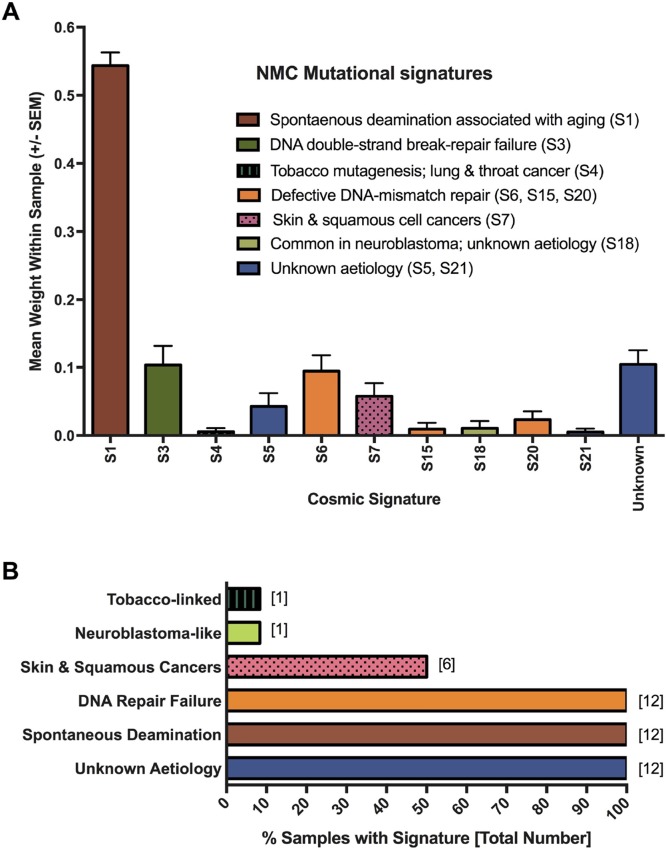
Mutagenesis signatures associated with NMC samples Signatures known to be associated with particular cancer types or mutagenic processes were extracted from MuTect-derived somatic SNV profiles. The contribution (weight) of each signature to the profile of each sample was averaged to produce the plot shown in panel (**A**). Panel (**B**) shows the proportion of NMC samples showing evidence of the indicated mutational processes.

To gain a better understanding of the mutagenic processes that underpin such genetic alterations, we next assessed the summarized nucleotide transition/transversion (Ti/Tv) ratios for each of the samples. The Ti/Tv ratios varied from an average of 2.10 in the NMC cell lines (range: 1.58–2.54) to 2.8 in the P896 primary sample, values that are similar to the WES estimates of ~2.8 from the 1000 Genome Project Consortium [45, 46]. However, this variation suggested that there may be important differences in the underlying mutagenic processes affecting these tumors. To examine this in greater detail, we applied the computational algorithm deconstructSigs [47] to extract previously reported mutational signatures from the Wellcome Trust Sanger Institute Mutational Signature Framework (http://cancer.sanger.ac.uk/cosmic/signatures) [43, 48, 49]. In total, 11 unique signatures were associated with the NMC samples (summarized in Figure 4A; results for individual samples are provided in Supplementary Figure 1). The single most prevalent signature (S1) was associated with an endogenous mutational process that is found in the majority of cancers, but there were several signatures associated with a failure of DNA-repair (S3, S6, S15 and S20; Figure 4A), with all NMC samples having at least one or more of these signatures (Figure 4B). It is likely that these two observations are related, with a defect in DNA-repair expected to lead to higher rates of background mutation. Signature S1 is the result of spontaneous deamination of 5-methylcytosine and is associated with small insertions and deletions, whereas signature S3, which is associated with failure of double-strand break-repair by homologous recombination, is associated with large (longer than 3bp) insertions and deletions with overlapping micro-homology at breakpoint junctions. Signatures S6, S15 and S20 all result from defective DNA mismatch repair and are associated with smaller indels at mono/polynucleotide repeats. Finally, half of the samples had a mutational signature (S7) associated with head, neck or oral squamous cell cancers (Figure 4B). Although the cell of origin of NMC is not yet known and the disease is not specific to the head or neck, it most closely resembles a poorly differentiated form of squamous carcinoma and historically has frequently been misdiagnosed as such [3, 50, 51].

### Recurrently mutated genes in NMC

**Figure 5:**
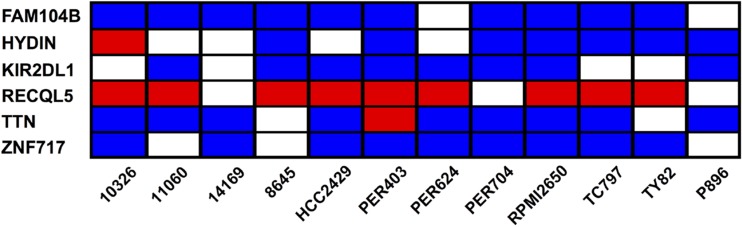
Genes carrying baseline deleterious variants in at least two-thirds of NMC samples Each individual sample may carry multiple variations in a single gene, and the heatmap summarizes the most severe type of variant associated with each given gene and sample. The color-coding represents the VEP annotation for biological impact: blue cells, moderate impact; red cells, high impact; blank cells, samples with no variation in the given gene.

We next aimed to identify common features within the mutational profile of the NMC samples. Taking the baseline deleterious variants shown in Figure 3A, we determined which genes were affected by at least one variant across multiple samples. None of the mutated genes carried variations in every NMC sample, but six genes (*FAM104B*, *HYDIN*, *KIR2DL1*, *RECQL5*, *TTN* and *ZNF717*) harbored variants in at least two-thirds of the NMC samples (Figure 5 and Supplementary DataFile3). Moreover, one recurring variant predicted by VEP annotation to have a high biological impact, was found in the DNA helicase gene *RECQL5* (Figure 5). This variant, which was observed in 9 out of the 12 NMC samples and confirmed via Sanger sequencing, represented a TG insertion at a predicted splice-acceptor site (17_73626919_-/TG; located at the end of intron 11) previously reported in a small number of hematopoietic and upper aerodigestive tract cancer samples (COSM127072). As a splice site mutation, precise effects on isoform expression are difficult to predict and are dependent on cellular context. However, there is substantial evidence for the critical role of *RECQL5* in DNA-damage response, tumorigenesis and sensitivity to drug treatment [52–64]. In the absence of constitutive samples for the NMC cell lines, we cannot definitely determine whether the observed mutation in *RECQL5* in each case is somatic or germline, but its detection in 75% of samples suggests that it may play an important role in NMC tumor development and disease progression.

### Commonly affected functional pathways in NMC

**Figure 6:**
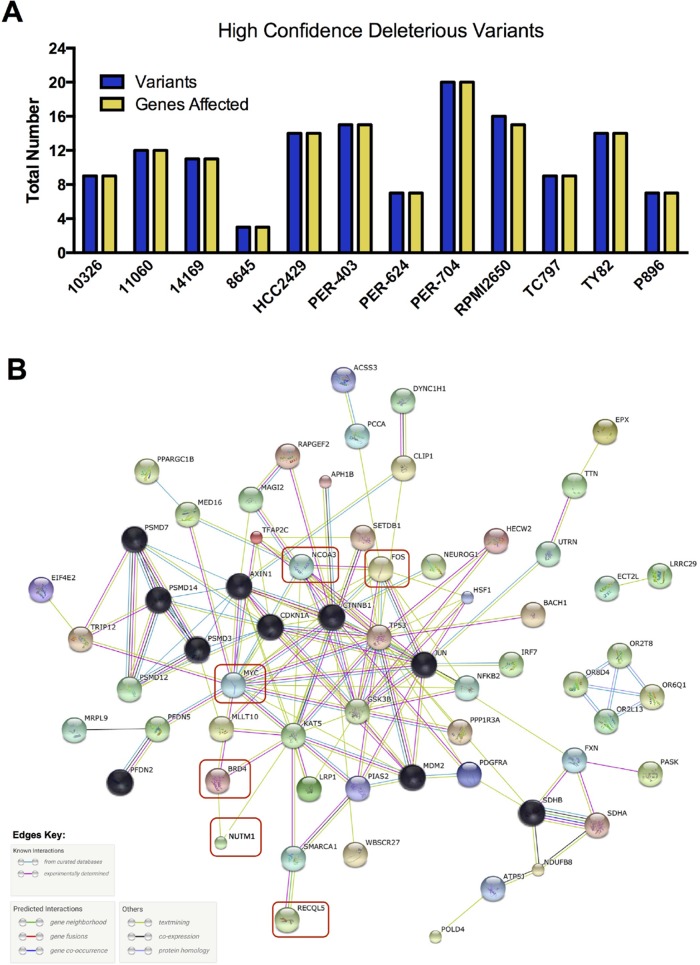
High confidence deleterious variants identified in individual NMC samples (**A**) Total number of variants and of genes affected by those variants. (**B**) Biological relationship of these high-confidence variants, along with additional genes of known relevance or mutational status (red boxes). Black nodes indicate those genes included by the STRING algorithm during network generation, with other nodes colored for visualization purposes only; the evidence used in generating connections (edges) between hubs is indicated in the key.

We next applied more stringent filtering to our baseline deleterious variant candidates, to identify the highest-impact variants for each sample in regard to predicted functional consequence (Figure 6A, Supplementary Figure 2 and Supplementary DataFile 4). The resulting 3–20 unique variants per sample were then analyzed with STRING v10.0 [65] to identify protein-protein interactions. After trimming orphan nodes, this resulted in a highly interconnected network (Figure 6B) with strong Gene Ontology enrichment for DNA-damage response, apoptosis, regulation of cell cycle and transcription, and the WNT-signaling pathway (false discovery rate < 0.0001 for all), in addition to a number of distinct cellular metabolic processes (full details in Supplementary DataFile4). KEGG pathways associated with several types of solid tumor were also significantly enriched, including those for basal cell carcinoma. The DNA-damage response signatures were related not only to the activity of RECQL5, but also the regulation of TP53, with this important cell-survival modulator being a central hub of the network (Figure 6B). Further investigation revealed that the *TP53* gene was affected by high-impact stop-gain SNV in TY82 (Supplementary DataFile 4), whilst HCC2429 carried a low-impact pathogenic missense mutation (Supplementary DataFile 3). Taken together, these data indicate that although each NMC sample may carry distinct exonic mutations, the affected genes appear to interact within a common biological network.

### Transcriptome profiling of NMC Samples

To further correlate the identified genetic variants with potential changes in biological function, we turned to next-generation transcriptome sequencing (RNA-Seq) to examine gene expression profiles across the cell line panel. Following RNA extraction and sequencing, we normalized RNA-Seq read counts to extract baseline gene expression estimates from NMC cell lines and the non-tumor line P896-FB (Supplementary DataFile 5), and looked for patterns of expression that could be correlated with either iBET resistance or cellular phenotype. Initially we interrogated a number of biologically relevant gene sets curated from the Reactome pathway database [66] and the GSEA Molecular Signatures database [67] (Supplementary Figure 4). While distinct clusters of genes with particularly low levels of expression, consisting mainly of histone-related transcripts, were evident among the chromatin-organization (Supplementary Figure 4A), cell cycle (Supplementary Figure 4C), and DNA-repair gene sets (Supplementary Figure 4E), and there were a small number of WNT-pathway genes that distinguished P896-FB from NMC lines (Supplementary Figure 4B), there was no obvious pattern of expression among these genes sets that could be readily correlated with either resistance profiles or NMC fusion-type.

We therefore restricted the analysis to focus on genes affected by high-confidence deleterious variants (i.e. those from Figure 6A). Unsupervised hierarchical clustering resulted in a clear separation of the fibroblast line P896-FB from the NMC samples as expected (Supplementary Figure 5), with expression of *NUTM1* being absent in this control line in contrast to the positive expression seen in the NMC lines. Expression of the neuroblastoma tumor suppressor gene *CASZ1* was also notably absent in P896-FB, in contrast to the NMC lines (Supplementary Figure 5). However, there were no obvious patterns in expression that correlated with the presence or absence of identified mutations in individual lines. For example, the expression levels of *SETDB1* in PER-403, which carried a frameshift indel, or *MED16* in PER-704, which carried a stop-gain SNV, were unremarkable compared to the other lines, and the expression of *RECQL5* was similar across all NMC lines. Hence, although these variants may affect the functionality of the affected transcripts, and differences may exist at the level of individual transcript expression, overall expression levels did not appear to be greatly affected for the majority of the variants detected. To address the potential expression of alternative transcripts of *RECQL5* in mutated lines, we examined exon-level read-counts from RNA-Seq, as well as isoform predictions for this gene returned using the Cufflinks suite of tools (http://cole-trapnell-lab.github.io/cufflinks). Using these methods, we were unable to detect differences in *RECQL5* splicing in lines carrying this variant, however it is important to remember that different splicing programs and factors may be brought to bear during differentiation, tumorigenesis, response to therapy, and other cellular contexts. As such, it is reasonable to suggest that differences in the transcriptional processing of *RECQL5* in mutated lines may only become evident under different cellular conditions or stages of differentiation.

### Genetic variations associated with poor iBET response

In an alternative approach to identify important genetic drivers of disease progression, we next investigated whether the attenuated response to iBET treatment that we observed in a subset of NMC cell lines (see Figure 2B) might correlate with the presence of specific genetic variants. Returning to the baseline deleterious exome variants, we looked for those that were absent in all of the iBET sensitive lines (14169, 10326, HCC2429, PER-403) but present in at least two of the three lines that responded poorly to iBET treatment (PER-624, RPMI2650, TC797). Three genes (*MUC6*, *IL11*, *NCOA3*) were found to carry moderate impact variants in RPMI2650 and PER-624 (Supplementary DataFile 5), but of these candidates, only the in-frame deletion in *NCOA3* (20_46279815_GCAGCAGCA/-) had obvious biological relevance for NMC. NCOA3 is a nuclear receptor co-activator with histone acetyltransferase activity that recruits p300/CBP and CREB binding protein as part of a multi-subunit co-activation complex. The identified variant has previously been reported in a single study in breast cancer (COSM1483713; rs751385560), and the deletion lies within a poly-glutamine (poly-Q) sequence in the carboxyl-terminal acetyl-transferase domain of the protein. Findings from Wong *et al*. suggest that a shorter poly-Q domain may increase the co-transactivation activity of NCOA3, potentially resulting in a more aggressive form of cancer [68]. Notably, of the four cell lines that had a mean iBET IC50 of > 1 μM, two carried the described *NCOA3* variant (RPMI2650 and PER-624), while the other two (TC797 and P896-CL) had significantly higher *NCOA3* expression compared to all other samples. In other carcinomas, high expression of this gene has been shown to be associated with tumor progression, metastasis and chemoresistance [69–71]. The data are therefore suggestive of an aberrant function for *NCOA3* in a subset of NMC cell lines, caused by either a deletion in the poly-Q domain or via an increase expression, that contributes to the attenuation of iBET cytotoxicity.

## DISCUSSION

NMC is an aggressive and currently incurable carcinoma with a characteristic *NUTM1* gene rearrangement. The considerable heterogeneity in patient age, tumor location and type of *NUTM1*-fusion expressed suggests that NMC may ultimately be divisible into clinically relevant sub-groups, with different clinical outcomes and treatment responses. To date however, only limited data are available to understand the potential genetic basis for such an approach. In a previous pilot study, we demonstrated that the response to iBETs varied considerably within a small panel of NMC cell lines, with one line being essentially unresponsive to treatment at physiologically relevant doses [21]. In the present study, we expanded on this observation using a significantly larger number of NMC samples and identified a distinct group that respond poorly to iBET treatment. Our analysis suggests that the type of *NUTM1*-fusion expressed may be one of the factors that contribute to iBET sensitivity, with cells expressing the *BRD4-NUTM1 ex11:ex2* fusion being more than ten-fold more responsive to iBET treatment on average than those with *BRD4-NUTM1 ex15:ex2* or *ex14:ex2* fusions. Changes in fusion-protein structure are likely to affect tertiary interactions with other regulatory molecules, histones, and drugs, as has been demonstrated for single-nucleotide bromodomain polymorphisms [72], and these differences in structure may thus be relevant for determining cytotoxic responses to iBETs. We also identified a highly significant correlation between iBET efficacy and sensitivity to iAURKs. This finding is consistent with a recent publication showing that iBET treatment directly suppresses the *AURKA* and *AURKB* genes in triple negative breast cancer cells [37]. *AURKB* has been reported to be a direct target of BRD4 [35], suggesting that the phenotype of the iBET-sensitive NMC cell lines may at least be partially dependent on AURK activity.

In accordance with the clinically observed heterogeneity of NMC, whole exome sequencing of these rare samples identified very few high-confidence mutations that were shared between independent specimens. However, we were able to identify a recurrent high-impact mutation in the gene *RECQL5*, a DNA helicase involved in interstrand crosslinking repair [55, 73]. *RECQL5* is essential for the maintenance of genomic stability, and polymorphisms in the gene have been associated with both poor prognosis in osteosarcoma and susceptibility to breast cancer [61, 62, 74, 75]. With deletion of the gene in mice also shown to increase cancer susceptibility, it is clear that *RECQL5* is an important tumor suppressor [76, 77]. Together with the background mutagenic signatures that we have described, as well as the biological network represented by additional high-impact mutations, our findings provide compelling evidence for a potential defect in the processes of DNA-repair within the genome of NMC cells. Based on these observations, it is possible that the mutation of *RECQL5* promotes the acquisition of additional mutations necessary for the NMC phenotype. Therapeutically, this type of genetic instability could potentially render NMC vulnerable to synthetic lethal interactions. Such an approach has been successfully exploited with the use of PARP inhibitors for the treatment of BRCA deficient ovarian cancer [78], wherein inhibition of PARP1 mediates mitotic catastrophe and apoptosis of BRCA deficient cells [79, 80]. A recent study has reported strong synthetic lethality between *RECQL5* and an activating V617F mutation in the *JAK2* tyrosine kinase in patients with myeloproliferative neoplasms, and it is possible that the JAK/STAT cascade may be worthy of further exploration in the context of NMC [63]. Interestingly, like *NUTM1* itself, the expression of *RECQL5* has been reported to be particularly high in the testis, indicating that there may be overlap in the transcriptional programs governing the expression of these two genes [81].

During the preparation of this manuscript, another group reported whole-genome sequencing of a small number of NMC samples, showing that complex chromosomal rearrangements, known as chromoplexy, may also be a recurring feature of this disease [82]. Although these authors did not find mutational signatures for defects in DNA-repair, the observation of catastrophic genome events in NMC is consistent with the potential failure of these pathways that we have described in the present study. Importantly, the somatic mutation rate the authors reported for NMC samples (1.1/Mb) is comparable to our estimates from the present study (1.4–3.5/Mb). Although the authors did not report the *RECQL5* variation we have described, their study was limited to only three NMC samples and further work will be required to assess the true prevalence of this mutation in the NMC population.

A limitation of the present study is the lack of matched normal samples for the purpose of variant calling; however, with NMC being one of the rarest cancers that exists, and less than 200 patients having so far been recorded around the world, very few primary samples are in fact available for such studies. To overcome this limitation, we have utilized a very conservative bioinformatic pipeline to filter normal population variants, and have focused only on those with the greatest potential impact for biological function. It is important to highlight therefore, that although we have only considered non-synonymous coding mutations, additional biological signals from other regions of the genome are also likely to contribute to NMC pathology. In the present study, we have highlighted the germline variant profile of a single NMC patient, cognizant of the fact that so few primary samples from this extremely rare tumor type exist around the world, however many more patient samples will be required to draw definitive conclusions about the involvement of germline mutations in the oncogenesis of NMC.

## MATERIALS AND METHODS

### Cells and cell culture

The NMC cell line P896-CL (also known as PER-909) and the matching non-tumor fibroblast line P896-FB (also known as PER-904N) were established from clinical biopsies of NMC patient P896 [25]. The NMC cell lines 14169 [31], 8645 [29] and 10326 [30] were grown in DMEM supplemented with 2 mM L-glutamine and 10% fetal calf serum (FCS). P896-FB was grown under the same conditions but with 15% FCS. P896-CL [25] and PER-891 were maintained in RPMI-1640 containing 2 mM L-glutamine, 10 nM 2-mercaptoethanol, 20% FCS, non-essential amino acids and pyruvate. All other NMC lines (HCC-2429 [22], PER-403 [23], PER-624 [24], PER-704 [21], TC797 [26], TY82 [27], RPMI2650 [28], and 11060 [10]) and the non-NMC carcinoma lines (PER-535 [21], PER-536, PER-712 and CAKI-1 [83]) were grown in RPMI-1640 containing the same supplements as for P896-CL and PER-891 but only 10% FCS. The following cell lines were kindly provided by external researchers: TC797 (JA Toretsky, Georgetown University, Washington DC, USA), 10326, 8645, 14169, 11060 (CA French, Brigham and Women's Hospital, Boston, USA), and HCC2429 (University of Texas Southwestern Medical Center, Dallas, USA). RPMI2650, TY82 and CAKI-1 were obtained from the DSMZ (German Collection of Microorganisms and Cell Cultures), the Japanese Collection of Research Bioresources Cell Bank (JCRB), and the American Type Culture Collection (ATCC) respectively, whilst A549, WI-38 and HS-27 were provided by the Children's Cancer Institute Drug Discovery Centre, Sydney, Australia. NUTM1-fusion breakpoints in NMC cell lines were confirmed by RT-PCR, followed by Sanger sequencing (see Supplementary Figure 6 and Supplementary Materials).

### Drug-screening assays

Drug screening was conducted at the Children's Cancer Institute Drug Discovery Centre, Sydney, Australia, and assessed the toxicity of 23 compounds in 12 NMC cell lines, six non-NMC carcinoma lines and two non-cancer fibroblast lines. Compounds were obtained from the following sources: docetaxel, vincristine sulfate, methotrexate, vorinostat, gefitinib, erlotinib, alisertib/MLN8237, AMG-900, epothilone B, JQ1, OTX-015 (MedChem Express, Monmouth Junction, NJ, USA); pyrvinium pamoate, digoxin, barasertib/AZD1152 (Sigma-Aldrich, St. Louis, Missouri, USA); I-BET151, GSK-2801 (GlaxoSmithKline, Brentford, UK); flavopiridol (Selleck Chemicals, Houston, TX, USA); PFI-1 (Sapphire Bioscience, Redfern, NSW, Australia); gemcitabine, daunorubicin, topotecan, mitoxantrone, monensin (Children's Cancer Institute Drug Discovery Centre, Sydney, NSW, Australia). Cells in log-phase growth were seeded in 96-well assay-ready plates using a Multidrop-384 (Thermo Scientific, Waltham, MA, USA) and incubated for 96 hours at 37°C in the presence of the indicated drug or vehicle (DMSO). Screening was performed with drug concentrations from 0.0025 μM to 10 μM (8-point serial dilutions), with IC50 values subsequently determined in two independent experiments. Response to drug treatment was determined by dispensing 10% (v/v) Alamar Blue reagent to assay plates using a Multidrop-384 (Thermo Scientific). Following a 6 hour incubation at 37°C metabolic activity was determined by measurement of fluorescence intensity (ex 555 nm, em 585 nm) using an EnSpire plate reader (PerkinElmer, Waltham, Massachusetts, USA). Percentage cell viability was calculated relative to positive and negative (vehicle only) controls. Data analysis included generation of dose response curves and calculation of IC50 values using the ActivityBase software suite (IDBS, Guildford, UK). Unsupervised hierarchical clustering of cell lines and drugs based on mean IC50 values was performed using the software Genesis [84].

### Whole-exome sequencing (WES)

Genomic DNA was isolated using the DNeasy Blood & Tissue Kit (Qiagen, Valencia, CA, USA) from 11 NMC cell lines (10326, 11060, 14169, 8645, HCC2429, PER-403, PER-624, PER-704, RPMI2650, TC797, TY82), as well as the primary sample P896 and the P896-FB fibroblast line grown from this patient. Whole exome 100bp paired-end sequencing (WES) was performed at AGRF (the Australian Genome Research Facility, Brisbane, QLD, Australia) using the Agilent SureSelect QXT Human All Exon +UTRs v5 (75Mb) target capture kit according to manufacturer's protocols, with samples multiplexed on the Illumina HiSeq 2500 platform. For details of the bioinformatic pipelines used for variant detection and the analysis of mutational signatures, please refer to the Supplementary Materials.

### Next-generation transcriptome sequencing (RNA-Seq)

Total RNA was isolated using TRIzol (Invitrogen, Mulgrave, VIC, Australia) and the RNeasy Mini Kit (Qiagen, Valencia, CA, USA) from 12 NMC cell lines (11060, 14169, 8645, RPMI2650, TC797, TY82, 10326, HCC2429, PER-403, PER-624, PER-704, P896-CL) and the P896-FB fibroblast line according to the manufacturers’ protocols, with QC performed using the Bioanalyzer 2100 (Agilent Technologies Australia, Mulgrave VIC). Sequencing libraries were prepared with Illumina's TruSeq stranded polyA protocols and were processed on an Illumina HiSeq 2000 (100bp paired-end sequencing) at AGRF (the Australian Genome Research Facility, Brisbane, QLD, Australia). For bioinformatic pipelines used for gene expression estimates and statistical analysis, please refer to the Supplementary Materials.

### Data availability

The raw data (BAM files) for all exome and transcriptome analyses performed in this study can be accessed from the NCBI's Short Read Archive (SRA), under Project Accession PRJNA339503 (SRA Accession SRP083924).

## SUPPLEMENTARY MATERIALS FIGURES AND TABLES













## Abbreviations

NMC: NUT midline carcinoma
iBET: bromodomain protein inhibitor
AURK: Aurora Kinase
WES: whole-exome sequencing
Ti/Tv: nucleotide transition/transversion ratio
P896-FB: fibroblast line derived from the P896 patient sample
P896-CL: cancer cell line derived from the P896 patient sample
