# Supplementary material for: Molecular-genetic profiling and high-throughput in vitro drug screening in NUT midline carcinoma—an aggressive and fatal disease
Source: Oncotarget. 2017 Dec 2;8(68):112313–29. doi: 10.18632/oncotarget.22862 (PMC5762512; doi:10.18632/oncotarget.22862)
Supplement: Supplementary file 1 [file oncotarget-08-112313-s001.pdf]

Molecular-genetic profiling and high-throughput *in vitro* drug screening in NUT midline carcinoma—an aggressive and fatal disease

SUPPLEMENTARY MATERIALS

Validation of NUTM1-translocation breakpoints in NMC cell lines

Total RNA was extracted in TRIzol (Life Technologies, Carlsbad, CA, USA), purified using the RNeasy Mini Kit (Qiagen, Valencia, CA, USA) and reverse transcribed using the SuperScript VILO cDNA Synthesis Kit (Life Technologies). Products were amplified using GoTaq Flexi DNA polymerase (Promega, Madison, WI, USA) and separated via agarose gel electrophoresis. PCR fragments were then purified with the QIAquick Gel Extraction Kit (Qiagen) and analyzed via Sanger sequencing at the Australian Genome Research Facility (AGRF, Nedlands, WA, Australia). Primer sequences used for variant detection were:

Cell lines	NUTM1-fusion	Forward primer	Reverse Primer
PER-403, 8645	BRD4-NUTM1 (ex11:ex2)	Eng_BRD4_Fwd_New (5′AAGTTGATGTGATTGCCGGCTCCTC-3′)	NUT_ex2_Rev_nest (5′CGGATATGAGCATGAAACCTAG-3′)
HCC-2425	BRD4-NUTM1 (ex11:ex2)	Eng_BRD4_Fwd_New (5′AAGTTGATGTGATTGCCGGCTCCTC-3′)	NUTex2-v1_647_R primer (5′-CTGATGCTCTCTGCTTTCCC-3′)
TC-797, TY-82	BRD4-NUTM1 (ex11:ex2) & (ex14:ex2)	Eng_BRD4_Fwd_New (5′AAGTTGATGTGATTGCCGGCTCCTC-3′)	NUT_ex2_Rev_nest (5′CGGATATGAGCATGAAACCTAG-3′)
PER-624, PER-704, RPMI-2650, 11060, 14169	BRD4-NUTM1 (ex15:ex2)	Ex15Fwd2 (5′AAGTTGATGTGATTGCCGGCTCCTC-3′)	NUT_ex2_Rev_nest (5′CGGATATGAGCATGAAACCTAG-3′)
10326	BRD3-NUTM1	Ex15Fwd2 (5′AAGTTGATGTGATTGCCGGCTCCTC-3′)	NUT_ex2_Rev_nest (5′CGGATATGAGCATGAAACCTAG-3′)
P896-CL	BRD4-NUTM1 (ex15:ex2Δnt1–585)	Ex15Fwd2 (5′AAGTTGATGTGATTGCCGGCTCCTC-3′)	NUTex3_v1_1300_R (5′GCAACTTTGACCGGATGATC-3′)

Whole-exome sequencing (WES), tumor-specific variant detection, annotation and filtering

Multiplexed whole exome 100 bp paired-end sequencing (WES) was performed on the Illumina HiSeq 2500 platform at AGRF (the Australian Genome Research Facility, Brisbane, QLD, Australia), using the Agilent SureSelect QXT Human All Exon plus UTRs v5 (75 Mb) target capture kit. Total data yield was ~5 Gb per sample (median of 93 million reads/sample), with >88% of bases having a sequence quality per base above Q30; conservative median depth coverage (excluding top 1% of outliers and accounting for PCR duplicates) was 85x in each sample, with >92% of targets having at least 20x coverage. Following de-multiplexing, raw fastq files were aligned to the hg19 human genome reference using bwa-mem [1]. To identify somatic and functionally relevant mutations, the resulting BAM files were processed in parallel through two different tools designed to handle paired-tumor normal samples–MuTect and Strelka [2, 3]. It is important to note that MuTect reports single nucleotide variations (SNVs) only, whereas Strelka reports both SNVs and indels. Since we did not have matched tumor-normal pairs for the majority of samples, we used the whole exome sequencing of the fibroblast cell line P896-FB (derived from the NMC patient P896), as a pseudo-normal control in each case. This assisted in filtering out non-specific variation associated with, for example, cell culturing or tissue of origin effects, as well any systematic artefacts specific to the particular exome sequencing platform used in this study (please note that P896-FB is referred to as PER-904N in the NCBI Short Read Archive record for this project).

MuTect (version 1.1.7) was run in HC (High Confidence) mode individually for each tumor (NMC sample or cell line) in comparison to P896-FB, using the hg19 human genome reference, COSMIC database v72 (Cosmic.hg19.v72.combined.vcf), and dbSNP_138 (dbsnp_138.hg19.vcf), restricting analysis to target regions (Agilent SureSelect Human All ExonV5 plus UTRs)

and filtering out 'REJECT' variant calls from the final VCF output. Strelka (version 1.0.14) was run in exome-sequencing mode (i.e. with depth filter disabled), again using P896-FB as the normal control for each sample, and filtering 'passed' variant calls for target regions. Subsequently we merged the MuTect and Strelka results (vcf files) to create the union of variants (both SNVs and indels) called by either method for each sample. VCF output files were then decomposed (to minimal representation of multi-allelic sites), normalized (left-aligned) and de-duplicated (to unique variants) using Vt [4], then annotated with Variant Effect Predictor (VEP) from Ensembl (ensembl-tools-release-82, with local assembly for GRCh37), using established Plugins for CADD (Combined Annotation Dependent Depletion, v1.3) [5] and ExAC (Exome Aggregation Consortium, v0.3) [6] databases.

To further assist in the elimination of germline variants from the raw MuTect/Strelka combined output, as well as identify those with potential functional significance, we took the VEP-annotated variant files (*.vep), and initially employed a conservative filtering approach to restrict variants to those occurring within protein-coding transcripts that also had CADD_PHRED scores > 10, minor allele frequencies <1% across all ExAC, 1000 Genome and dbSNP146 populations, and a minimum VEP-predicted functional impact of either 'moderate' or 'high'; variants that had PolyPhen scores of either 'benign' or 'unknown' *and* SIFT scores for 'tolerated' or 'low-confidence' were also filtered out. Annotations were restricted to a single transcript per variant by selecting those predicted to be the most severe by VEP (i.e. PICK = 1), and by prioritizing canonical transcripts. The candidates from these minimally-filtered vep files we refer to as *baseline deleterious variants*. To generate lists of *high-confidence deleterious variants* associated with each cell line or sample, additional filtering was used; specifically, by selecting only those with a predicted functional impact of 'HIGH' and which either had a known COSMIC annotation or were novel (i.e. were not otherwise annotated in dbSNP146).

Somatic mutation rate and analysis of mutational signatures

For the purpose of calculating somatic mutation rates and extracting mutational signatures, we returned to the raw variant output from MuTect and filtered out all commonly occurring variants (those with minor allele frequencies >1% in any of the ExAC and 1000 Genome populations, or dbSNP146), and those with a CADD_PHRED score (predicted deleteriousness) of <20. Somatic mutation rates were then derived by dividing the resulting variant counts by the full target region of the exome platform used for sequencing (i.e. 75 Mb, which includes both coding and UTR regions). Transition/transversion (Ti/Tv) ratios were calculated from these SNVs using the VariantEval tool from the Genome Analysis Toolkit (GATK 2.8 data bundle for hg19). To derive mutational signatures for each sample, these SNV profiles were analyzed using the R-based tool deconstructSig [7], in conjunction with the pre-installed library of COSMIC signatures provided with the package (R version 3.1.3; deconstructSig v1.6.0), and using the 'default' method of normalization for trinucleotide counts across the genome.

Germline variant analysis

To identify potential germline variants associated with the primary NMC sample P896 that would have been overlooked during the paired analysis described above, we performed a separate variant calling pipeline on all 13 samples using the Genome Analysis Toolkit (GATK) [8], according to GATK Best Practice recommendations [9, 10]. Using the GATK 2.8 data bundle for hg19, we thus performed base quality score recalibration, indel realignment, duplicate removal, SNV/indel discovery and genotyping, across all samples simultaneously, then applied empirically determined hard-filtering parameters to remove false positives and restrict variants to target capture regions (for SNVs: MQ < 40, MQRankSum < –12.5, ReadPosRankSum < –8, DP < 20, QUAL < 30, QD < 2, FS > 60; for indels: MQ < 60, ReadPosRankSum < –20, DP < 20, QUAL < 30, QD < 2, FS > 200). To assist with the filtering of common variants and/or sequencing artefacts in this pipeline, we also downloaded whole exome sequencing data from six normal human blood samples published by Meienberg *et al*. that were processed on the same platform used in the present study (Agilent SureSelect QXT Human All Exon plus UTRs v5 target capture with Illumina sequencing) [11]. Each of these samples were sequenced by two different vendors (V1 and V2), resulting in a total of 12 fastq files that were downloaded and aligned to the hg19 human genome reference using bwa-mem, prior to performing variant calling and filtering with GATK as described above.

To identify potential germline variants carried by the P896 NMC sample, we next selected all variants that were present in *both* P896 and its corresponding fibroblast cell line (P896-FB), but which were *absent* in all 12 normal samples from the study by Meienberg *et al* [11]. Finally, we annotated these variants using VEP and further filtered them using the same conservative criteria described earlier to identify tumor-specific 'baseline deleterious variants', with the purpose of removing common population variants and those with little likelihood of functional impact. The surviving variants represented the final shortlist of potentially functional germline variants associated with P896, and their GATK-derived genotypes were subsequently determined for all NMC samples.

Next-generation transcriptome sequencing (RNA-seq) and analysis

Sequencing libraries were prepared with Illumina's TruSeq stranded polyA protocols and processed on an Illumina HiSeq 2000 (100bp paired-end sequencing) at AGRF (the Australian Genome Research Facility, Brisbane,

QLD, Australia); QC metrics indicated that >87% bases had quality scores greater than Q30 across all samples. Tophat2 (v2.0.13) was used to map reads to the hg19 human genome reference before summarizing gene read-counts using htseq-count (HTSeq framework v0.6.0) in reverse-strand mode and specifying a minimum alignment quality score of 20 [12]. RefSeq annotations for GRCh37/hg19 (Feb. 2009 assembly) used in this pipeline were downloaded from UCSC (http://genome.ucsc.edu/cgi-bin/hgTables) in refFlat GTF format, and filtered to remove redundant chromosome annotations ("hap", "ChrUn" and "Random"). The resulting raw gene counts were normalized for gene length and GC content using the CQN package (conditional quantile normalization) from Bioconductor [13] and a constant, empirically determined sizeFactor for all samples (i.e. without adjustment for library size at this stage); hg19 gene lengths and GC content were calculated in R using the GenomicRanges, rtracklayer and Rsamtools packages. The resulting expression estimates were then normalized for differences in sample library size using the DESeq2 package [14] with levels set to match individual samples, extracting final baseline gene expression values for each cell line using the counts() function and confirming the effect of normalization using relative log expression (RLE) plots (Supplementary Figure 3). These final values thus represent the baseline (resting) summarized expression values for hg19 genes in each sample.

To identify the genes with significantly different levels of expression between iBET sensitive & poorly responding NMC cell lines, we took the CQN pre-processed expression estimates (as above) and again passed them to DESeq2, this time with the two groups (i.e. sensitive vs. poor responders) used to define the analysis levels ($n = 4$ sensitive lines; $n = 3$ resistant lines). The genes returned are those significantly different between resistant/sensitive lines (high values signifying high expression in resistant lines), with p-values adjusted for multiple testing according to the method of Benjamini-Hochberg ("padj", the default DESeq2 output).

Variant validation by sanger sequencing

Genomic DNA was amplified by nested PCR using LongAmp DNA polymerase (New England BioLabs Inc., Ipswich, MA, USA), purified using the QIAquick Gel Extraction Kit (Qiagen, Valencia, CA, USA) and Sanger sequenced at the Australian Genome Research Facility (AGRF, Nedlands, WA, Australia). The primers used for different targets are provided in the Table below:

Primer	Sequence (5′ to 3′)
RECQL5_exon10_for	GGATGAAGGCAGAGATGAGG
RECQL5_exon13_rev	CATCAGCGGTACGTGTTGAC
NCOA3_exon19_for	CCTACTGCTGGTGGTGCTG
NCOA3_exon20_rev	TAGGAGGTGGGCTGAAGG
P53_exon7_for_HCC2429	TGGCTCTGACTGTACCACCA
p53_exon8_rev_HCC2429	GTGAGGCTCCCCTTTCTTG
P53_exon9_for_TY82	CCAACAACACCAGCTCCTCT
P53_exon10_rev_TY82	CTGGGCATCCTTGAGTTCC
SMARCA1 _exon4_for	TGTCTGAGAGTCGGAAAACATC
SMARCA1 _exon6_rev	AACCATGTGAGGTCCAGGAA

REFERENCES

1. Li H, Durbin R. Fast and accurate short read alignment with Burrows-Wheeler transform. Bioinformatics. 2009; 25:1754–1760.
2. Cibulskis K, Lawrence MS, Carter SL, Sivachenko A, Jaffe D, Sougnez C, Gabriel S, Meyerson M, Lander ES, Getz G. Sensitive detection of somatic point mutations in impure and heterogeneous cancer samples. Nat Biotechnol. 2013; 31:213–219.
3. Saunders CT, Wong WS, Swamy S, Becq J, Murray LJ, Cheetham RK. Strelka: accurate somatic small-variant calling from sequenced tumor-normal sample pairs. Bioinformatics. 2012; 28:1811–1817.
4. Tan A, Abecasis GR, Kang HM. Unified representation of genetic variants. Bioinformatics. 2015; 31:2202–2204.
5. Kircher M, Witten DM, Jain P, O'Roak BJ, Cooper GM, Shendure J. A general framework for estimating the relative pathogenicity of human genetic variants. Nat Genet. 2014; 46:310–315.
6. Lek M, Karczewski KJ, Minikel EV, Samocha KE, Banks E, Fennell T, O'Donnell-Luria AH, Ware JS, Hill AJ, Cummings BB, Tukiainen T, Birnbaum DP, Kosmicki JA, et al. Analysis of protein-coding genetic variation in 60,706 humans. Nature. 2016; 536:285–291.
7. Rosenthal R, McGranahan N, Herrero J, Taylor BS, Swanton C. DeconstructSigs: delineating mutational processes in single tumors distinguishes DNA repair deficiencies and patterns of carcinoma evolution. Genome Biol. 2016; 17:31.
8. McKenna A, Hanna M, Banks E, Sivachenko A, Cibulskis K, Kernytsky A, Garimella K, Altshuler D, Gabriel S, Daly M, DePristo MA. The Genome Analysis Toolkit: a MapReduce framework for analyzing next-generation DNA sequencing data. Genome Res. 2010; 20:1297–1303.
9. DePristo MA, Banks E, Poplin R, Garimella KV, Maguire JR, Hartl C, Philippakis AA, del Angel G, Rivas MA, Hanna M, McKenna A, Fennell TJ, Kernytsky AM, et al. A framework for variation discovery and genotyping using next-generation DNA sequencing data. Nat Genet. 2011; 43:491–498.
10. Van der Auwera GA, Carneiro MO, Hartl C, Poplin R, Del Angel G, Levy-Moonshine A, Jordan T, Shakir K, Roazen D, Thibault J, Banks E, Garimella KV, Altshuler D, et al. From FastQ data to high confidence variant calls: the Genome Analysis Toolkit best practices pipeline. Curr Protoc Bioinformatics. 2013; 43:11.10.11–11.10.33.
11. Meienberg J, Zerjavic K, Keller I, Okoniewski M, Patrignani A, Ludin K, Xu Z, Steinmann B, Carrel T, Rothlisberger B, Schlapbach R, Bruggmann R, Matyas G. New insights into the performance of human whole-exome capture platforms. Nucleic Acids Res. 2015; 43:e76.
12. Anders S, Pyl PT, Huber W. HTSeq—a Python framework to work with high-throughput sequencing data. Bioinformatics. 2015; 31:166–169.
13. Hansen KD, Irizarry RA, Wu Z. Removing technical variability in RNA-seq data using conditional quantile normalization. Biostatistics. 2012; 13:204–216.
14. Love MI, Huber W, Anders S. Moderated estimation of fold change and dispersion for RNA-seq data with DESeq2. Genome Biol. 2014; 15:550.

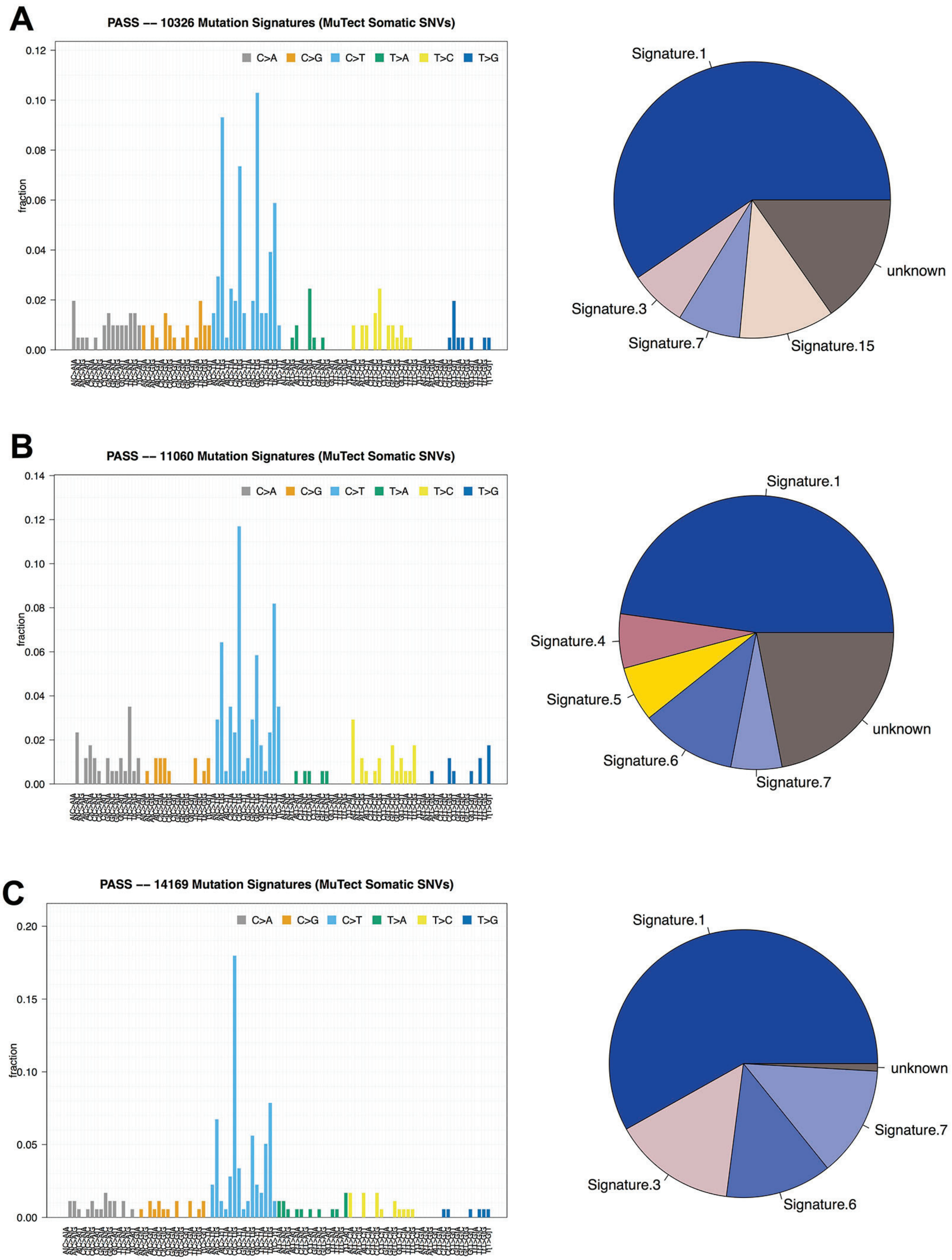
A
PASS -- 10326 Mutation Signatures (MuTect Somatic SNVs)
C>A
C>G
C>T
T>A
T>C
T>G
fraction
0.00
0.02
0.04
0.06
0.08
0.10
0.12
Signature.1
Signature.3
Signature.7
Signature.15
unknown
B
PASS -- 11060 Mutation Signatures (MuTect Somatic SNVs)
C>A
C>G
C>T
T>A
T>C
T>G
fraction
0.00
0.02
0.04
0.06
0.08
0.10
0.12
0.14
Signature.1
Signature.4
Signature.5
Signature.6
Signature.7
unknown
C
PASS -- 14169 Mutation Signatures (MuTect Somatic SNVs)
C>A
C>G
C>T
T>A
T>C
T>G
fraction
0.00
0.05
0.10
0.15
0.20
Signature.1
unknown
Signature.7
Signature.6
Signature.3

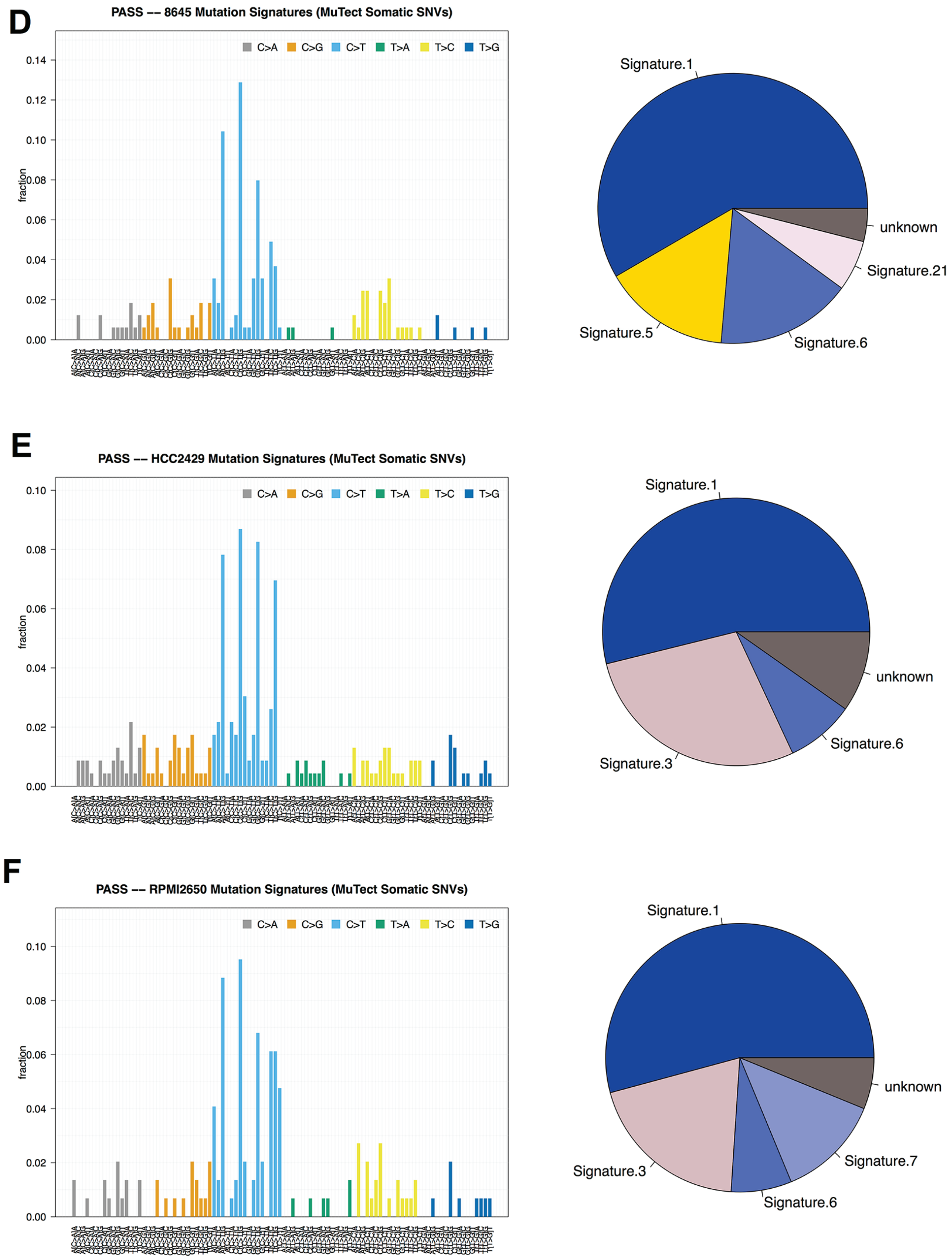
D
PASS -- 8645 Mutation Signatures (MuTect Somatic SNVs)
C>A
C>G
C>T
T>A
T>C
T>G
fraction
0.14
0.12
0.10
0.08
0.06
0.04
0.02
0.00
Signature.1
unknown
Signature.21
Signature.6
Signature.5
E
PASS -- HCC2429 Mutation Signatures (MuTect Somatic SNVs)
C>A
C>G
C>T
T>A
T>C
T>G
fraction
0.10
0.08
0.06
0.04
0.02
0.00
Signature.1
unknown
Signature.6
Signature.3
F
PASS -- RPMI2650 Mutation Signatures (MuTect Somatic SNVs)
C>A
C>G
C>T
T>A
T>C
T>G
fraction
0.10
0.08
0.06
0.04
0.02
0.00
Signature.1
unknown
Signature.7
Signature.6
Signature.3

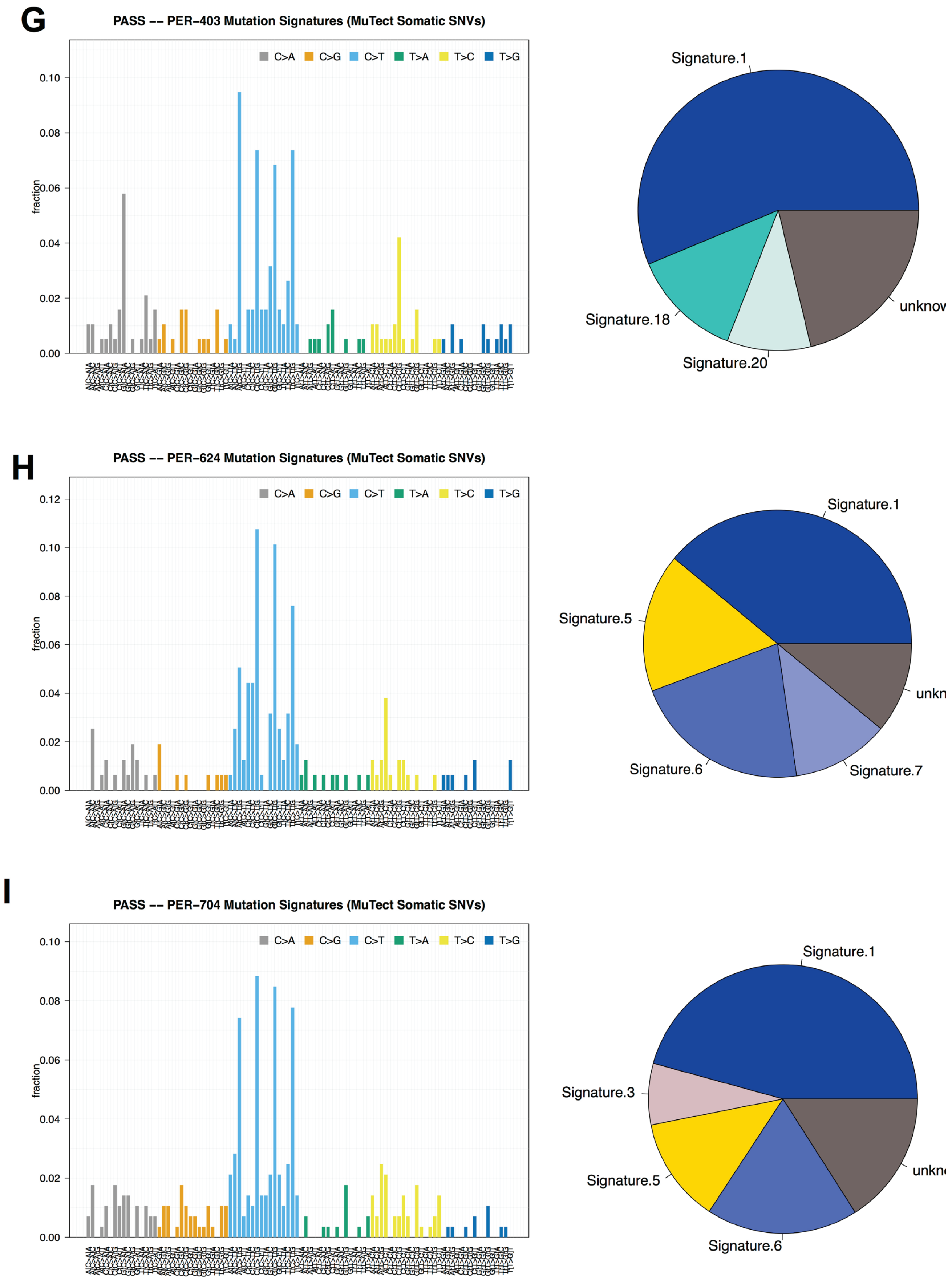
G
PASS –– PER–403 Mutation Signatures (MuTect Somatic SNVs)
C>A
C>G
C>T
T>A
T>C
T>G
fraction
0.00
0.02
0.04
0.06
0.08
0.10
Signature.1
Signature.18
Signature.20
unknov
H
PASS –– PER–624 Mutation Signatures (MuTect Somatic SNVs)
C>A
C>G
C>T
T>A
T>C
T>G
fraction
0.00
0.02
0.04
0.06
0.08
0.10
0.12
Signature.1
Signature.5
Signature.6
Signature.7
unkn
I
PASS –– PER–704 Mutation Signatures (MuTect Somatic SNVs)
C>A
C>G
C>T
T>A
T>C
T>G
fraction
0.00
0.02
0.04
0.06
0.08
0.10
Signature.1
Signature.3
Signature.5
Signature.6
unkn

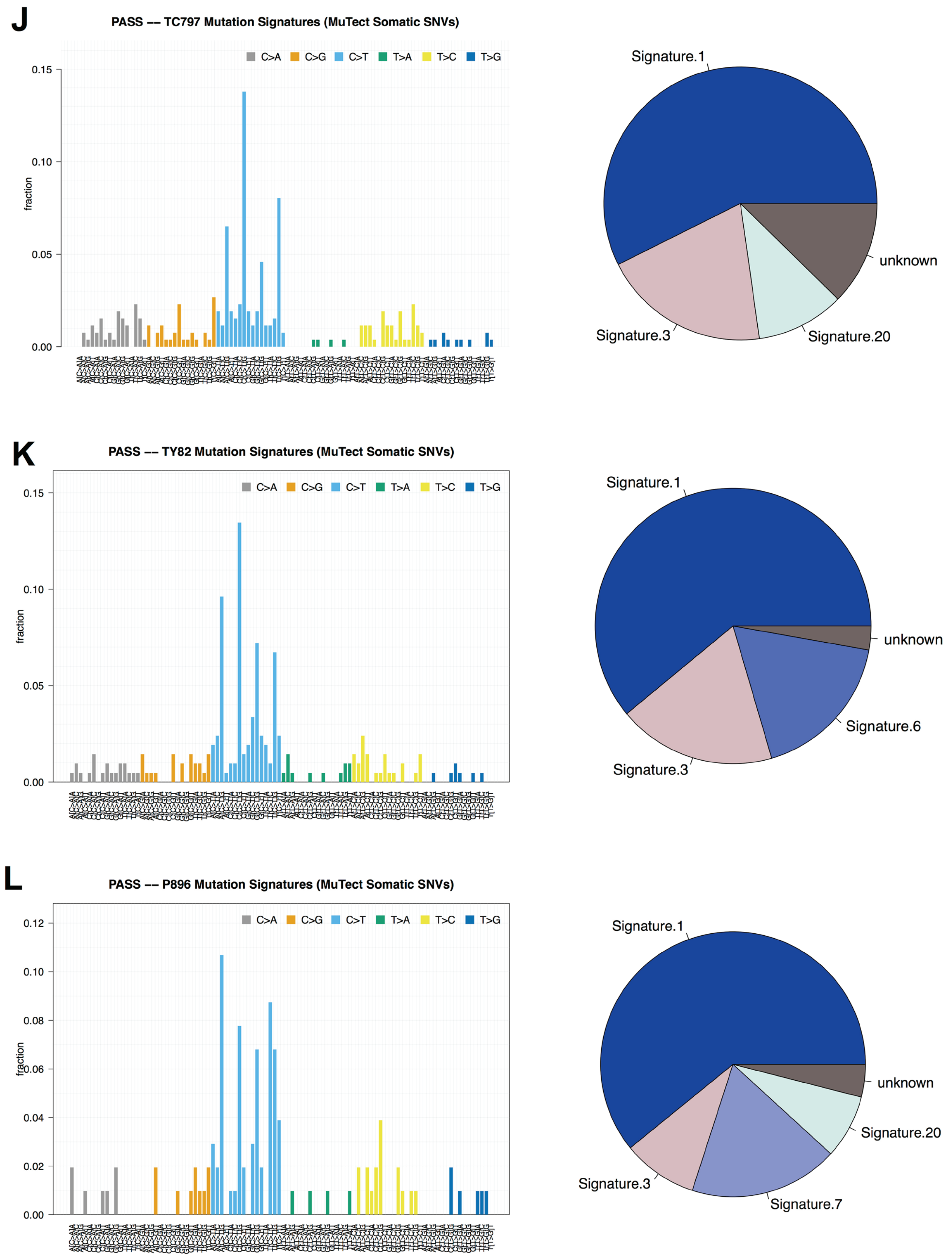
J
PASS -- TC797 Mutation Signatures (MuTect Somatic SNVs)
C>A C>G C>T T>A T>C T>G
fraction
0.15
0.10
0.05
0.00
Signature.1
unknown
Signature.20
Signature.3
K
PASS -- TY82 Mutation Signatures (MuTect Somatic SNVs)
C>A C>G C>T T>A T>C T>G
fraction
0.15
0.10
0.05
0.00
Signature.1
unknown
Signature.6
Signature.3
L
PASS -- P896 Mutation Signatures (MuTect Somatic SNVs)
C>A C>G C>T T>A T>C T>G
fraction
0.12
0.10
0.08
0.06
0.04
0.02
0.00
Signature.1
unknown
Signature.20
Signature.7
Signature.3

M

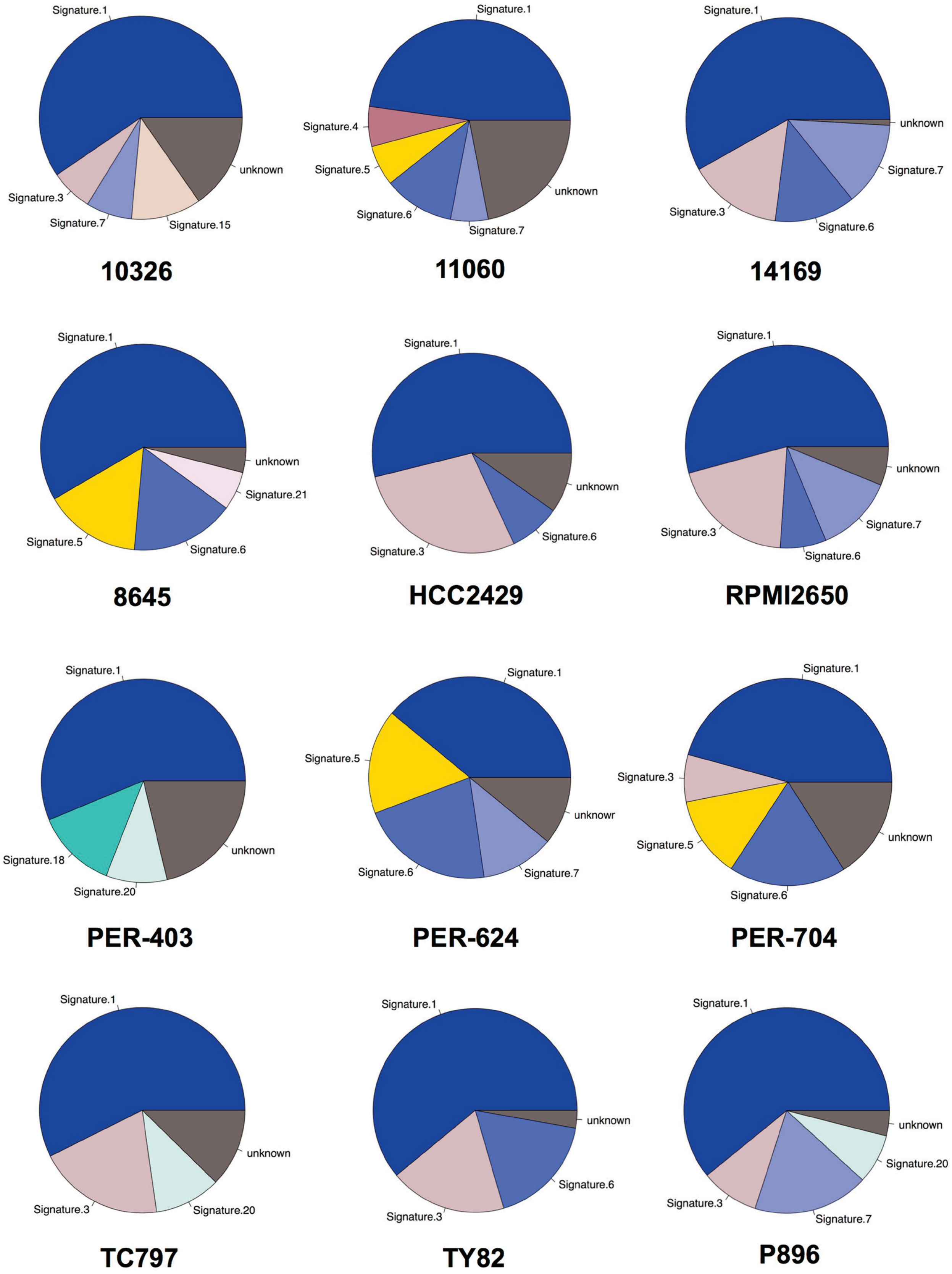

Signature.1
unknown
Signature.15
Signature.7
Signature.3
10326
Signature.1
Signature.4
Signature.5
Signature.6
Signature.7
unknown
11060
Signature.1
unknown
Signature.7
Signature.6
Signature.3
14169
Signature.1
unknown
Signature.21
Signature.6
Signature.5
8645
Signature.1
unknown
Signature.6
Signature.3
HCC2429
Signature.1
unknown
Signature.7
Signature.6
Signature.3
RPMI2650
Signature.1
unknown
Signature.20
Signature.18
PER-403
Signature.1
Signature.5
unknowr
Signature.7
Signature.6
PER-624
Signature.1
Signature.3
Signature.5
Signature.6
unknown
PER-704
Signature.1
unknown
Signature.20
Signature.3
TC797
Signature.1
unknown
Signature.6
Signature.3
TY82
Signature.1
unknown
Signature.20
Signature.7
Signature.3
P896

N

Sample	S1	S3	S4	S5	S6	S7	S15	S18	S20	S21	Unknown
10326	0.60	0.07				0.07	0.11				0.15
11060	0.48		0.06	0.07	0.11	0.06					0.22
14169	0.58	0.15			0.13	0.13					0.01
8645	0.58			0.15	0.16					0.06	0.04
HCC2429	0.54	0.28			0.08						0.10
PER-403	0.56							0.13	0.10		0.21
PER-624	0.39			0.17	0.22	0.12					0.11
PER-704	0.46	0.07		0.13	0.18						0.16
RPMI2650	0.54	0.20			0.07	0.13					0.06
TC797	0.57	0.20							0.10		0.12
TY82	0.61	0.19			0.18						0.03
P896	0.61	0.09				0.18			0.08		0.04

Supplementary Figure 1: Mutational signatures associated with each NMC sample. (**A–L**) Panels showing the base substitution frequencies profile (left) used to deconvolute the mutational signatures present within each sample (right). (**M**) Summary of the pie charts shown in previous panels. (**N**) Table detailing the calculated weights (or relative contribution) of each signature in each sample, that were used to generate the graphs shown in Figure 4 of the main manuscript.

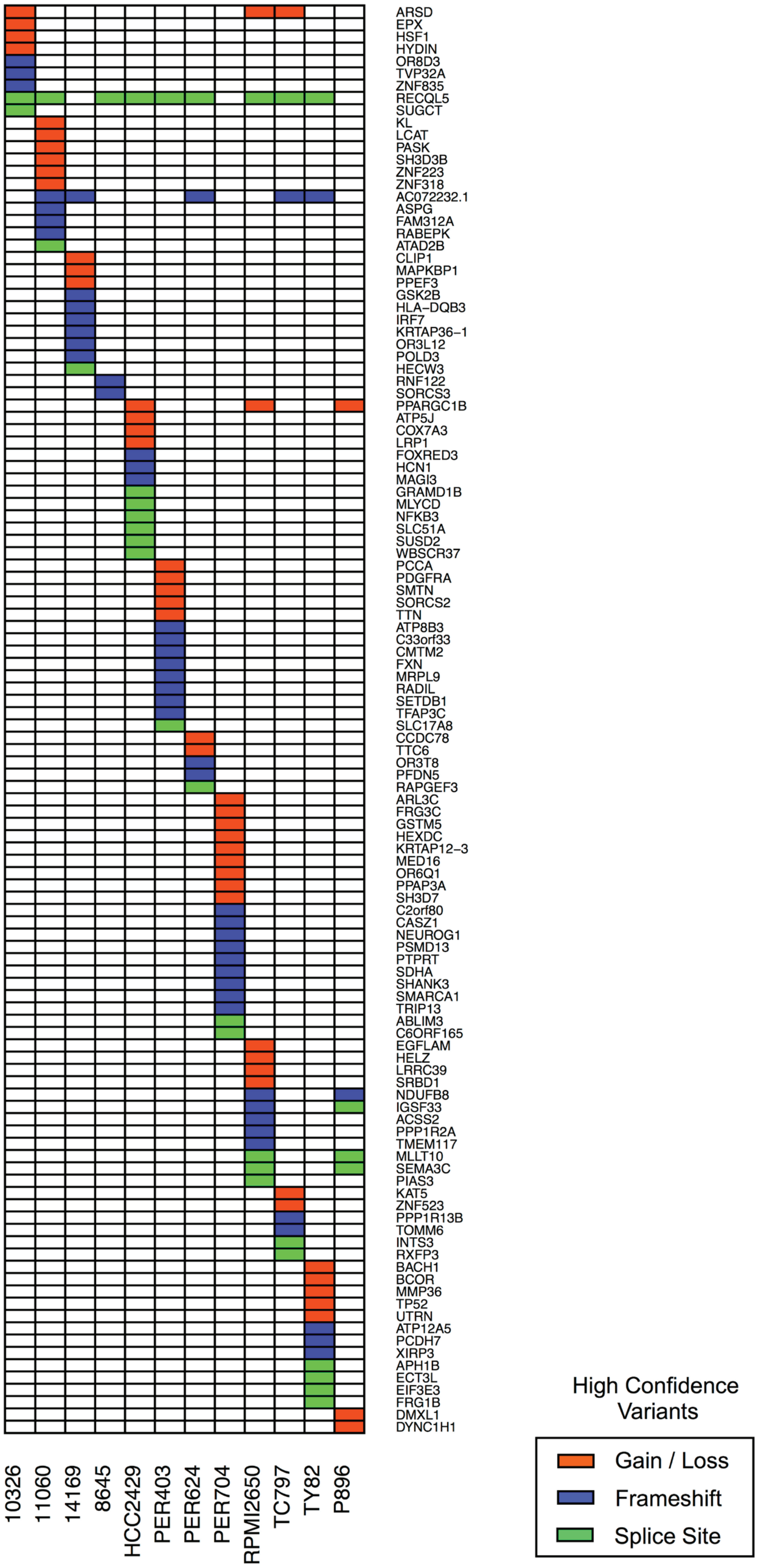

Supplementary Figure 2: Heatmap showing distribution of high-confidence deleterious gene variants across NMC samples. Red cells, variants causing either gain or loss of a start or stop codon (SNVs); blue cells, frameshift variants (indels); green cells, variants coinciding with acceptor or donor splice sites (SNVs or indels).

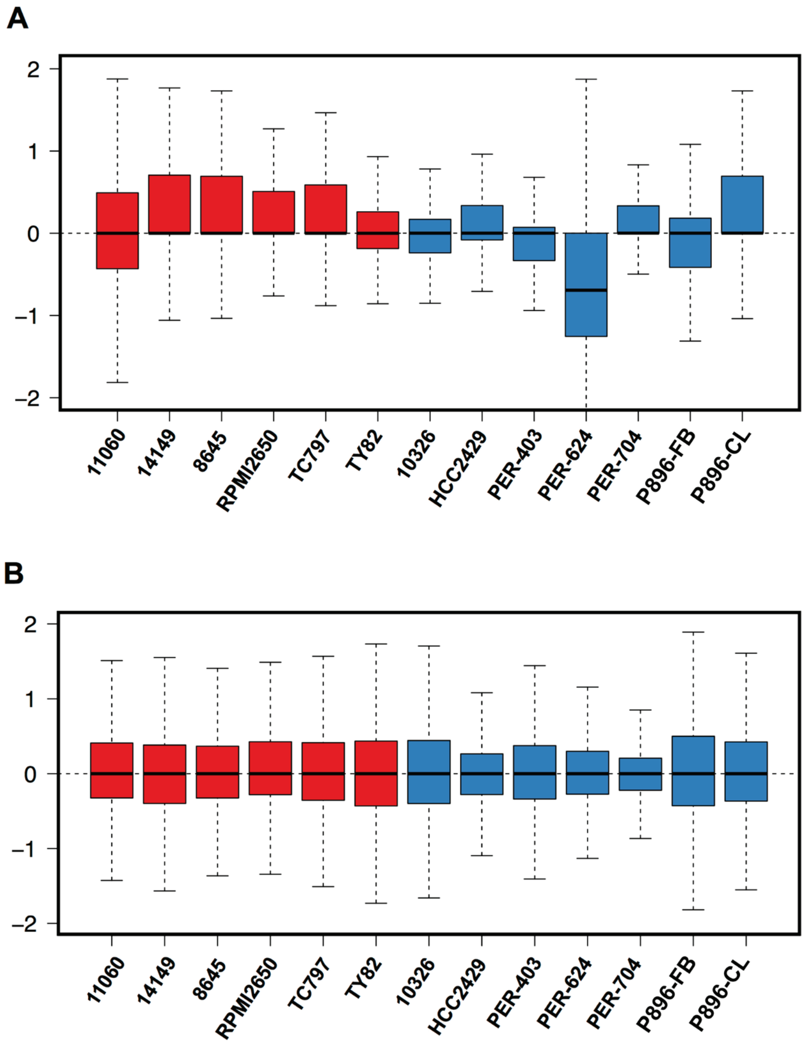

Supplementary Figure 3: Normalization of RNA-seq expression estimates. NMC sample libraries were prepared and sequenced in two batches (colored bars); plots show RLE (relative log expression) of read count data before (**A**) and after (**B**) normalization using CQN and DESeq2.

A

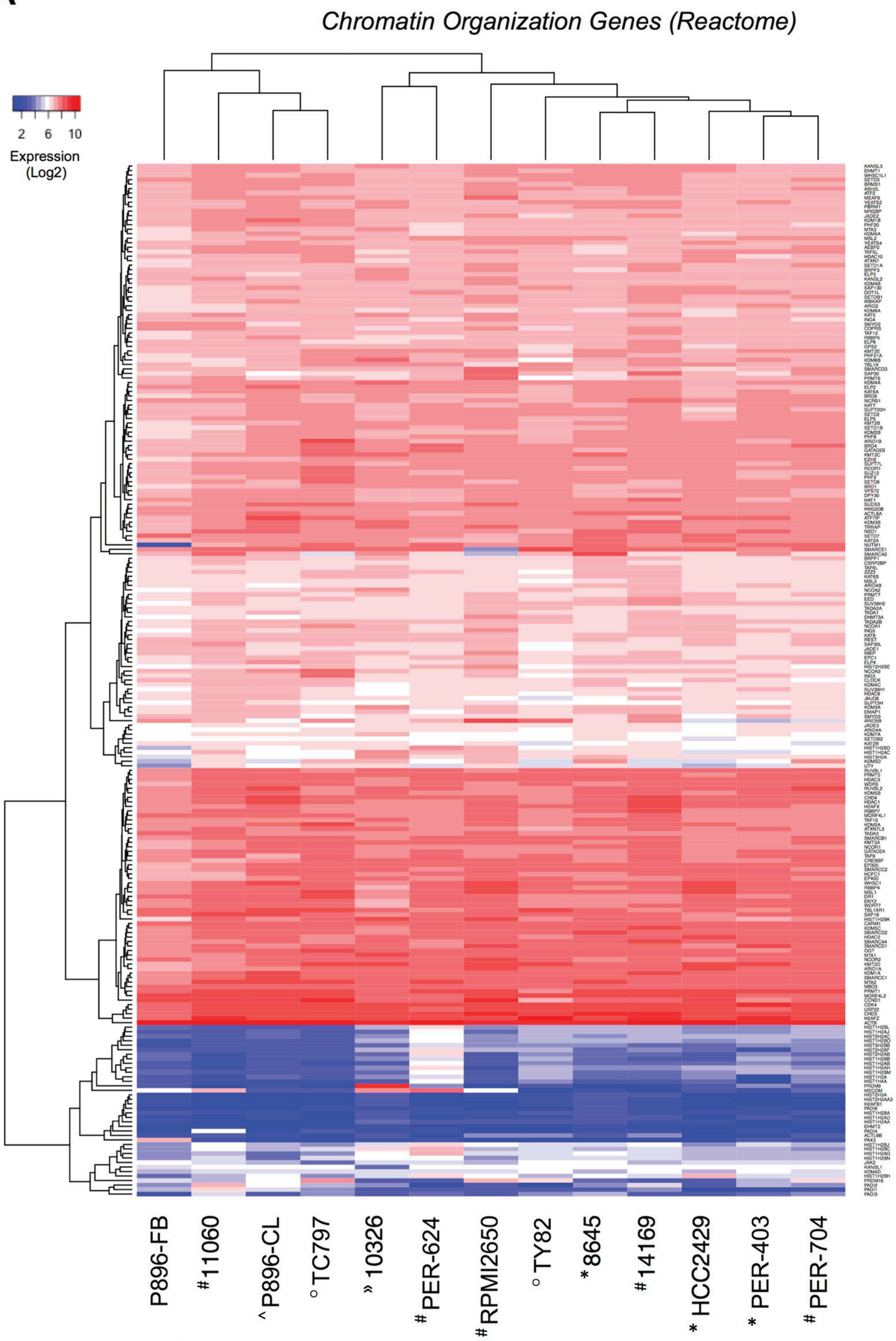

NUTM1-Translocation Key

BRD4-NUTM1 *[ex11:ex2], ◦[ex14:ex2], #[ex15:ex2], ^[ex15:ex2Δnt1–585]; and »[BRD3-NUTM1]

B

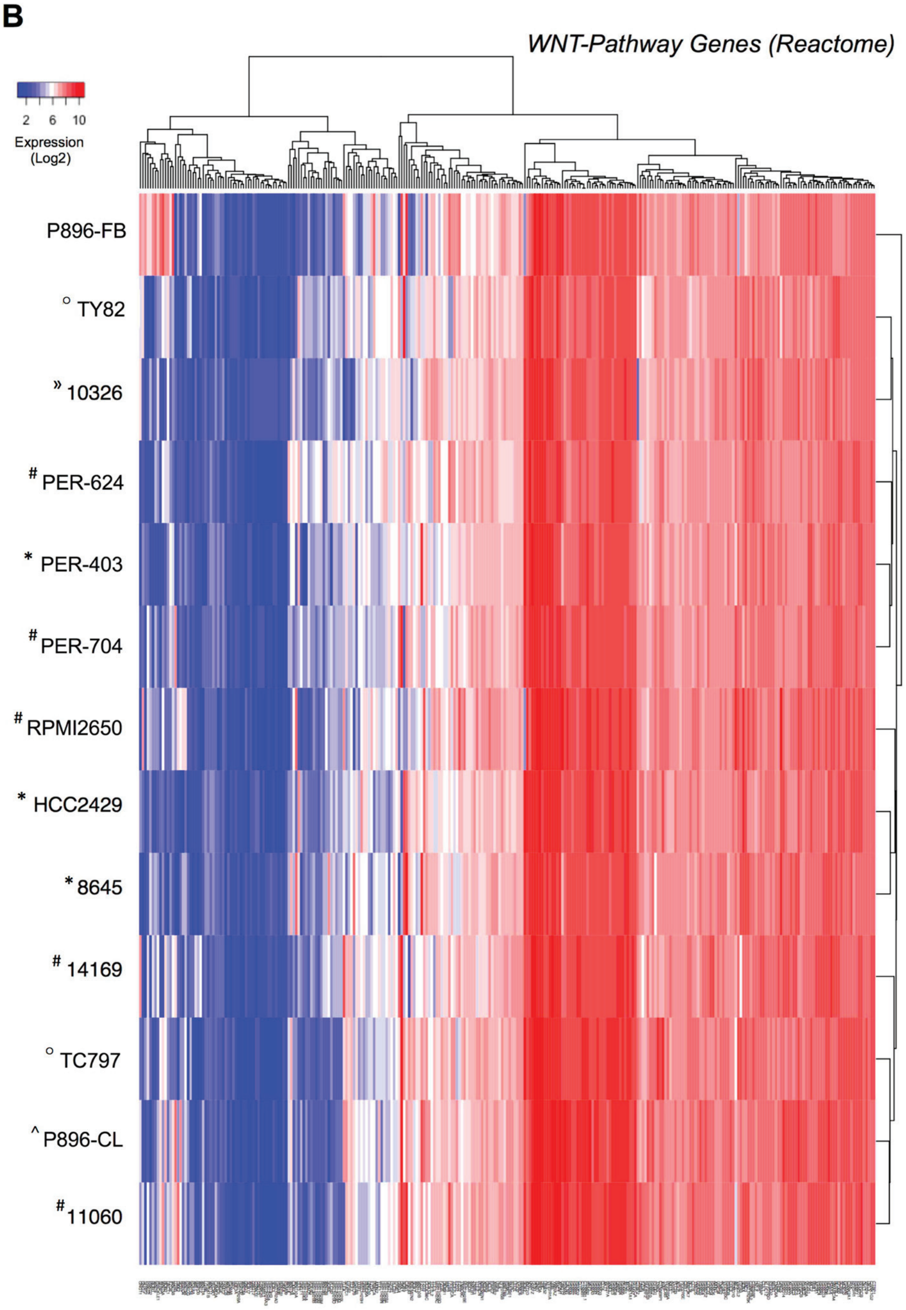

NUTM1-Translocation Key

BRD4-NUTM1 *[ex11:ex2], ◦[ex14:ex2], #[ex15:ex2], ^[ex15:ex2Δnt1–585]; and »[BRD3-NUTM1]

C

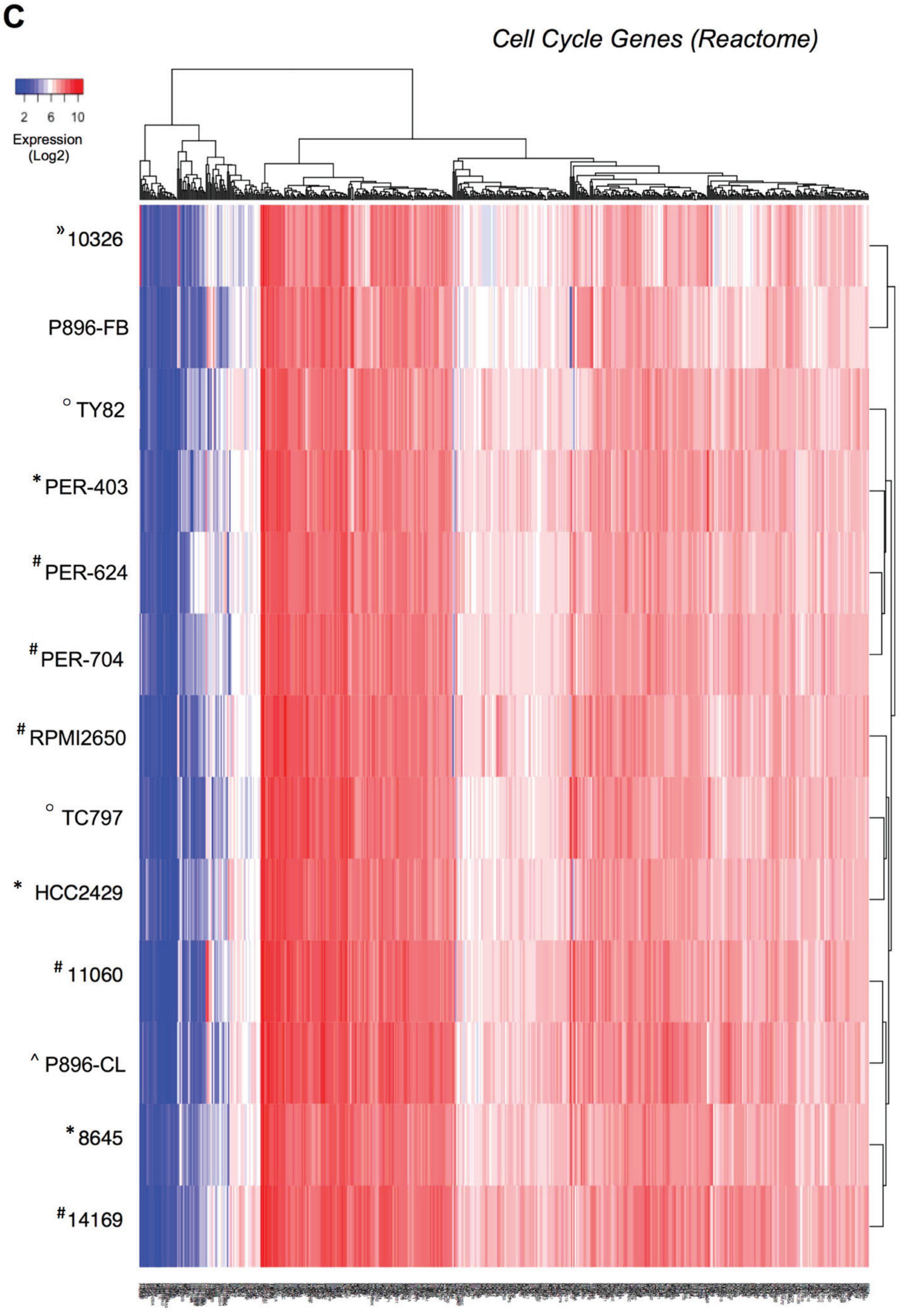

NUTM1-Translocation Key

BRD4-NUTM1 *[ex11:ex2], ◦[ex14:ex2], #[ex15:ex2], ^[ex15:ex2Δnt1–585]; and »[BRD3-NUTM1]

D

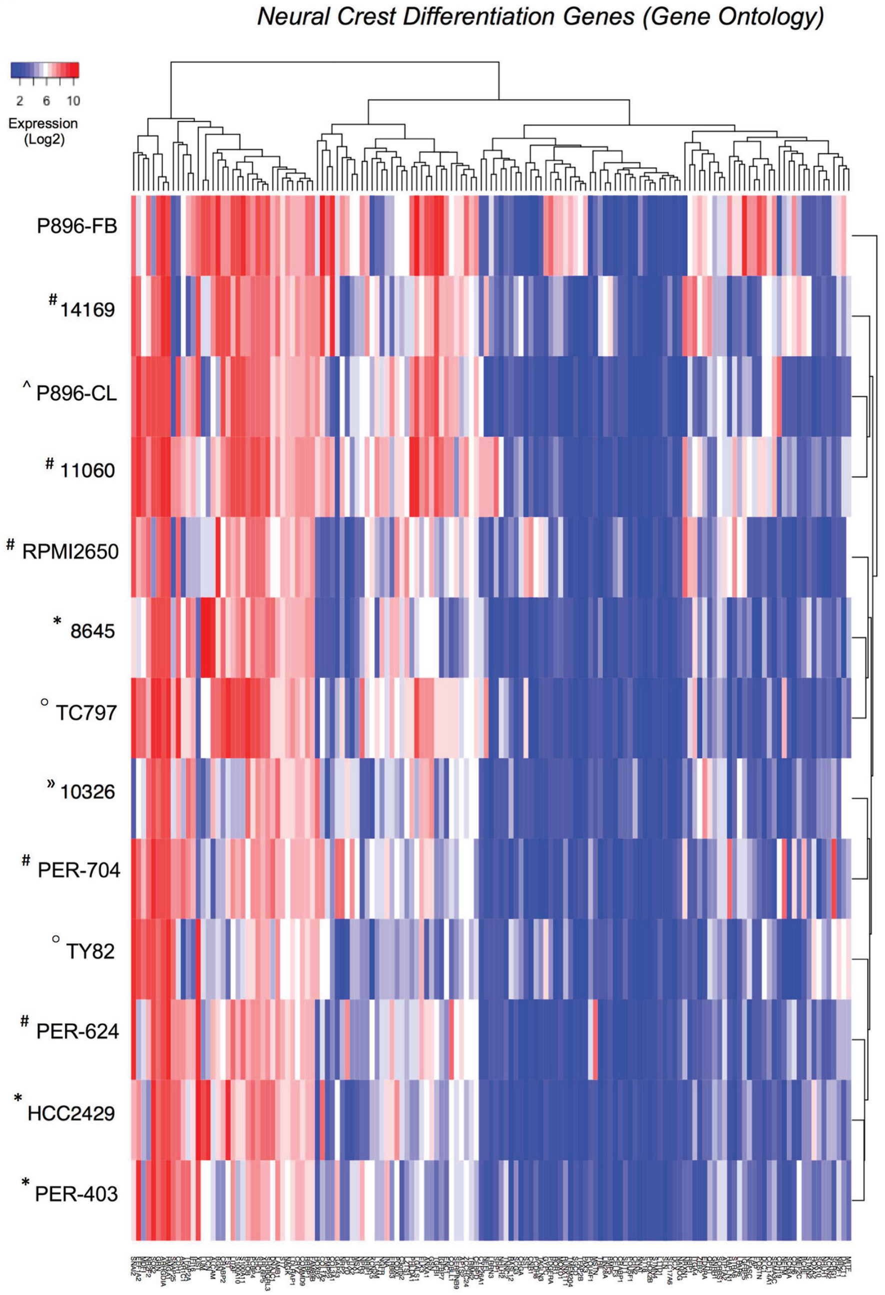

NUTM1-Translocation Key

BRD4-NUTM1 *[ex11:ex2], ◦[ex14:ex2], #[ex15:ex2], ^[ex15:ex2Δnt1–585]; and »[BRD3-NUTM1]

E

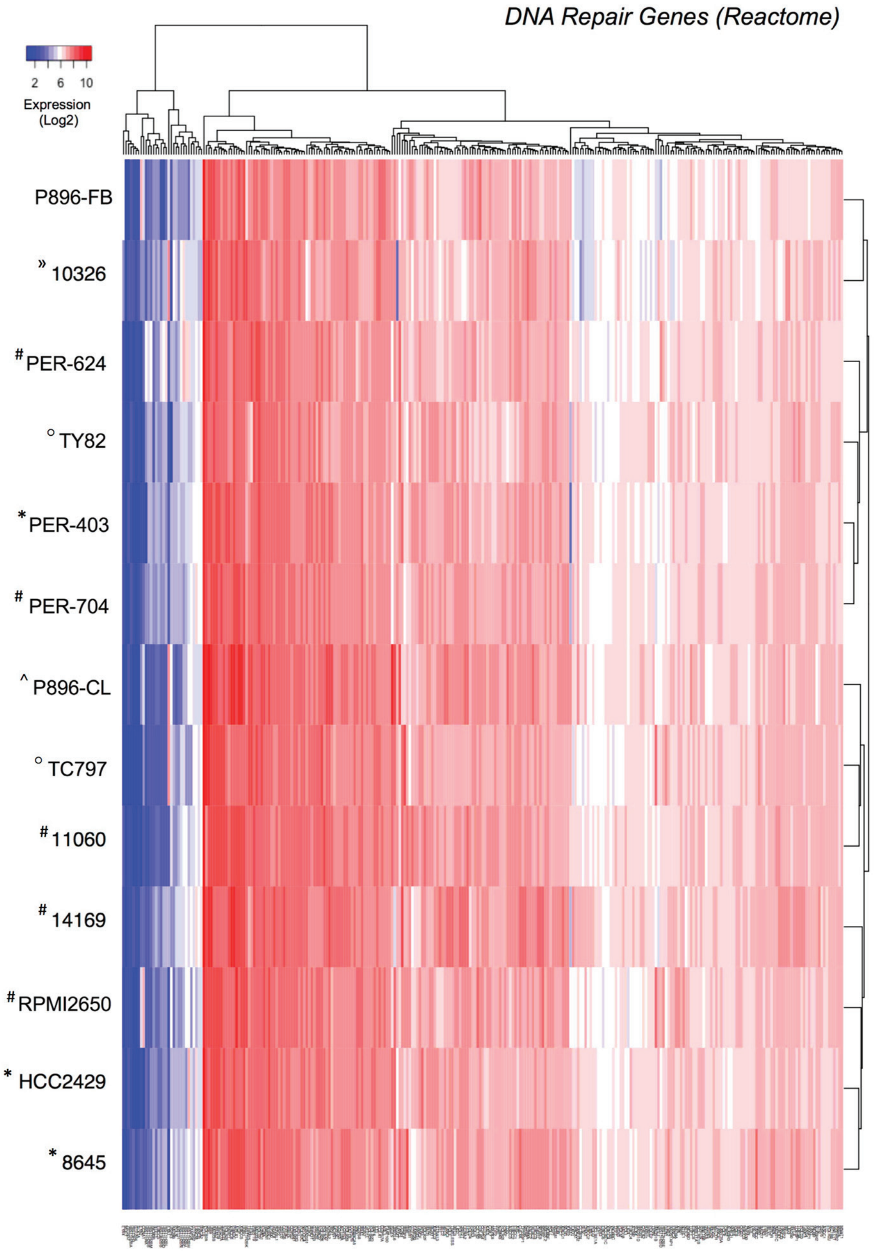

NUTM1-Translocation Key

BRD4-NUTM1 *[ex11:ex2], ◦[ex14:ex2], #[ex15:ex2], ^[ex15:ex2Δnt1–585]; and »[BRD3-NUTM1]

F

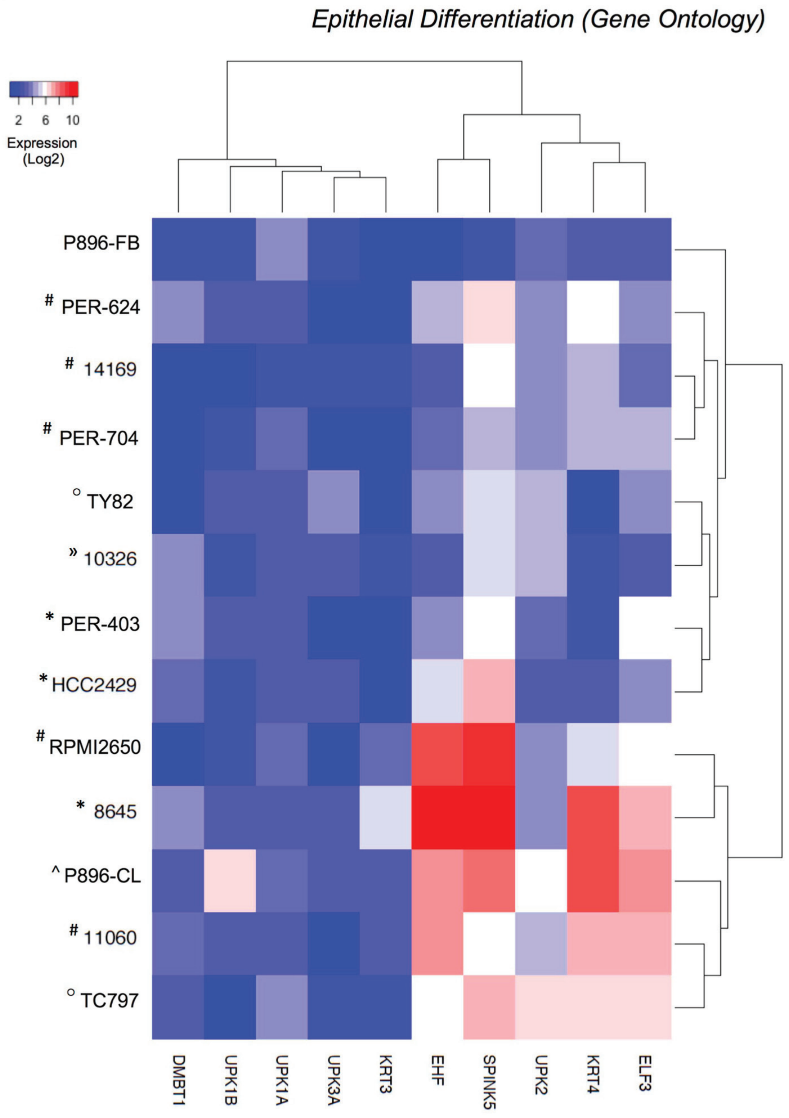

NUTM1-Translocation Key

BRD4-NUTM1 *[ex11:ex2], ∘[ex14:ex2], #[ex15:ex2], ^[ex15:ex2Δnt1–585]; and »[BRD3-NUTM1]

Supplementary Figure 4: Heatmap and unsupervised hierarchical clustering of mRNA expression estimates for genes involved with (**A**) chromatin organization, (**B**) the WNT-pathway, (**C**) cell cycle, (**D**) neural-crest differentiation, (**E**) DNA repair, and (**F**) epithelial differentiation. Gene lists were compiled from the Reactome Pathway Database v57 or using Gene Ontology terms. Data represent log2 normalized read counts derived from RNA-seq data. The different NUTM1-fusion variants expressed in the NMC cell lines are indicated with the following prefixes: BRD4-NUTM1 *[ex11:ex2], °[ex14:ex2], #[ex15:ex2], ^[ex15:ex2Δnt1–585]; and »[BRD3-NUTM1].

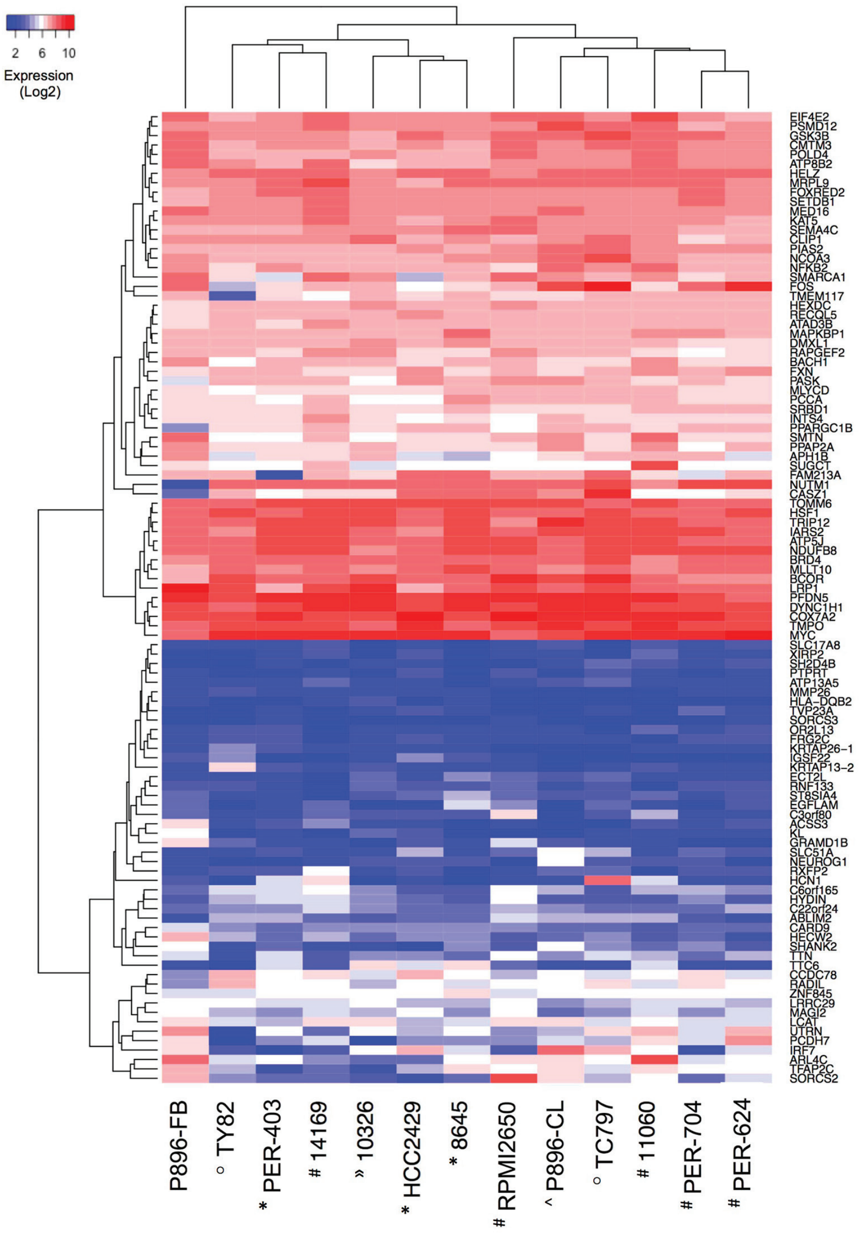

NUTM1-Translocation Key

BRD4-NUTM1 *[ex11:ex2], ∘[ex14:ex2], #[ex15:ex2], ^[ex15:ex2Δnt1–585]; and »[BRD3-NUTM1]

Supplementary Figure 5: Expression levels of high confidence deleterious variants. Heatmap and unsupervised hierarchical clustering of mRNA expression estimates for genes affected by high confidence variants in NMC samples. Data represent log2 normalized read counts derived from RNA-seq. The different NUTM1-fusion variants expressed in the NMC cell lines are indicated with the following prefixes: BRD4-NUTM1 *[ex11:ex2], °[ex14:ex2], #[ex15:ex2], ^[ex15:ex2Δnt1–585]; and »[BRD3-NUTM1].

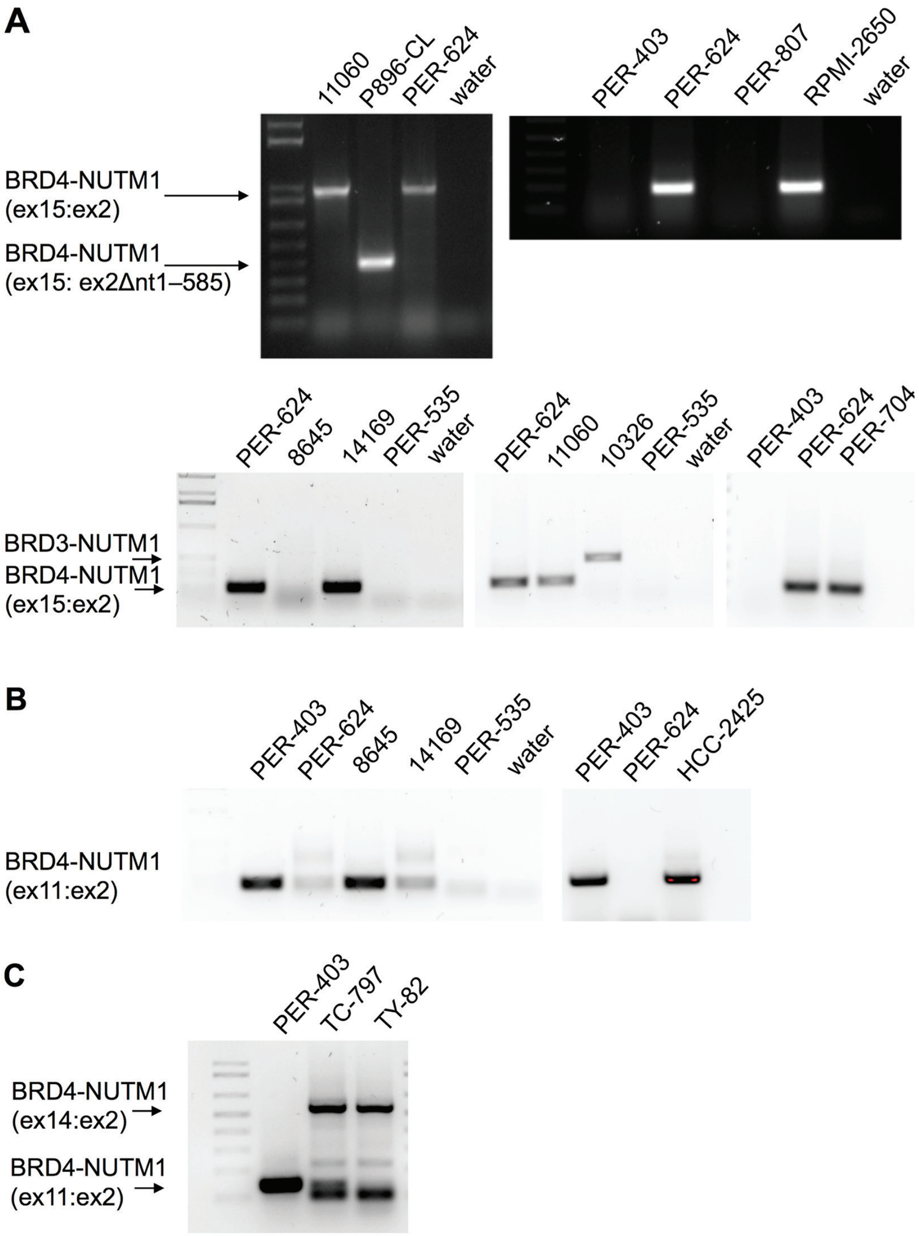

Supplementary Figure 6: NUTM1-fusion breakpoint validation by RT–PCR. (**A**) BRD4-NUTM1 (ex15:ex2) mRNA transcripts were detected in the NMC cell lines 11060 (top left panel), PER-624 (top left), RPMI-2650 (top right), 14169 (lower left), and PER-704 (lower right). The same primers detected expression of BRD4-NUTM1 (ex15:ex2Δnt1–585) and BRD3-NUTM1 in P896-CL (top left) and 10326 (lower middle panel), respectively. (**B**) BRD4-NUTM1 (ex11:ex2) fusions were present in the NMC lines PER-403, 8645 (left panel), and HCC-2425 (right panel). (**C**) TC-797 and TY-82 predominately express BRD4-NUTM1 (ex14:ex2) fusion transcripts, but also express (ex11:ex2) fusions at low levels, as previously reported. All NUTM1-fusion PCR products were confirmed by Sanger sequencing.

Supplementary Table 1: Recent/ongoing clinical trials involving NMC therapy

Study title	Study type	Study status as of June 2017	Class of therapeutic Agent	Patients enrolled as of June 2017	Study outcome	Clinical trial identifier/reference
A Study to Investigate the Safety, Pharmacokinetics, Pharmacodynamics, and Clinical Activity of GSK525762 in Subjects with NUT Midline Carcinoma (NMC) and Other Cancers	Prospective Clinical Trial	Recruiting	BET inhibitor	–	–	NCT01587703
Open Label, Multi-center Study to Assess the Safety, Tolerability and Pharmacokinetics of CUDC-907 in Subjects with Advanced/Relapsed Solid Tumors	Prospective Clinical Trial	Recruiting	PI3K/HDAC inhibitor	–	–	NCT02307240
A Phase 1/2, Open-Label Safety and Tolerability Study of INCB057643 in Subjects with Advanced Malignancies	Prospective Clinical Trial	Recruiting	BET inhibitor	–	–	NCT02711137
A Two-Part Study of RO6870810. Dose-Escalation Study in Participants with Advanced Solid Tumors and Expansion Study in Participants with Selected Malignancies	Prospective Clinical Trial	Active, not recruiting	BET inhibitor	–	–	NCT01987362
An Open-Label, Dose-Escalation Study of INCB054329 in Patients with Advanced Malignancies	Prospective Clinical Trial	Active, not recruiting	BET inhibitor	69	–	NCT02431260
A Dose-Finding Study of OTX105/MK-8628, a Small Molecule Inhibitor of the Bromodomain and Extra-Terminal (BET) Proteins, in Adults with Selected Advanced Solid Tumors (MK-8628-003)	Prospective Clinical Trial	Complete	BET inhibitor	47	–	NCT02259114
Clinical Response of Carcinomas Harboring the BRD4-NUT Oncoprotein to the Targeted Bromodomain Inhibitor OTX015/MK-8628	Clinical Proof of Concept Case Series	Published	BET inhibitor	4	MK-8628 monotherapy in pretreated NMC patients resulted in rapid response with tumor regression and symptomatic relief in two patients, disease stability in one patient and progressive disease in one patient. All four patients ultimately died.	*Stathis A, Zucca E, Bekradda M et al. Cancer Discovery 2016
Intensive Treatment and Survival Outcomes in NUT Midline Carcinoma of the Head and Neck Clinicopathologic Features and Long-term Outcome of NUT Midline Carcinoma	Retrospective International NMC Registry Clinical Review	Published	Multimodal therapy including surgery, radiotherapy, chemotherapy	92	NUT Midline Carcinoma has a very poor prognosis with conventional therapeutic modalities. Aggressive initial surgical resection significantly associated with enhanced survival. Radiotherapy may have an adjuvant therapeutic role. No statistical benefit for chemotherapy shown.	*Chau NG, Hurwitz S, Mitchell CM et al. Cancer 2016 *Bauer DE, Mitchell CM, Strait KM et al. Clin Cancer Res 2012

Supplementary DataFile 1: Clinical and molecular characteristics of NMC patients and cell lines. Also provided are the *in vitro* sensitivity results (IC50 values) for 23 drugs tested in the full cell line panel (12 NMC lines, six non-NMC carcinoma lines, and two control fibroblast lines). See Supplementary_DataFile_1

Supplementary DataFile 2: Germline variants associated with the NMC patient P896. A total of 179 rare variants with the potential for functional impact were common to P896 and the matched fibroblast line P896-FB, but absent in the exome sequences from normal human blood samples downloaded from a previously published study (samples 280V1, 280V2, 2905V1, 2905V2, 326V1, 326V2, 44V1, 44V2, 7344V1, 7344V2, 7739V1, and 7739V2). The genotype of these 179 variants was also assessed in the NMC cell lines. Genotypes are provided as: [Genotype of variant i.e. 0/0, homozygous REF (yellow shading); 0/1, heterozygous REF/ALT (pink shading); 1/1, homozygous ALT (green shading)]_[No. of exome sequencing reads for REF allele],[No. of exome sequencing reads for ALT allele]. See Supplementary_DataFile_2

Supplementary DataFile 3: Baseline deleterious variants associated with NMC samples. The full details of each variant are provided for every NMC sample or cell line, along with a summary of the variants common between samples. Six genes harbored variants in at least two-thirds of the NMC samples, as summarized in the heatmap. Baseline deleterious variants were defined as those that were rare in the normal population, affected protein-coding genes, and were predicted to have at least a moderate probability of biological impact. See Supplementary_DataFile_3

Supplementary DataFile 4: High-confidence deleterious variants associated with NMC samples. These variants represent the subset of baseline deleterious variants with the highest-impact in regard to predicted functional consequence. High-confidence variants from all NMC samples were pooled and analyzed with STRING to identify protein interaction networks, which were subsequently analyzed for enrichment of Gene Ontology (GO) and KEGG pathway terms. Variants (e.g. *RECQL5* and *TP53*) and GO enrichment terms (e.g. the Wnt pathway, and DNA-damage response pathways) of particular interest are highlighted. See Supplementary_DataFile_4

Supplementary DataFile 5: NMC gene expression profiles and molecular features associated with iBET resistance. Shown are the baseline gene expression estimates for NMC lines and the non-tumor line P896-FB, derived using RNA-Seq data (refer to Supp. Methods). Analysis revealed 388 genes with significant differences in expression between iBET sensitive (n =4) and poorly-responding NMC lines (n = 3), after correction for multiple testing ($p < 0.05$, Benjamini-Hochberg adjusted p-value (padj); positive fold-change indicates higher expression in resistant lines). Finally, we identified all baseline deleterious exome variants that were absent in iBET-sensitive lines (14169, 10326, HCC2429, PER-403) but present in at least two of the three lines that responded poorly to iBETs (PER-624, RPMI2650, TC797). Three genes (*MUC6*, *IL11*, *NCOA3*) were found to carry moderate-impact variants in the resistant lines RPMI2650 and PER-624, with the in-frame deletion in *NCOA3* having the greatest biological relevance for NMC. See Supplementary_DataFile_5
